# Prospects for Clinical Development of Stat5 Inhibitor IST5-002: High Transcriptomic Specificity in Prostate Cancer and Low Toxicity In Vivo

**DOI:** 10.3390/cancers12113412

**Published:** 2020-11-18

**Authors:** Cristina Maranto, Vindhya Udhane, Jing Jia, Ranjit Verma, Gerhard Müller-Newen, Peter S. LaViolette, Michael Pereckas, Lavannya Sabharwal, Scott Terhune, Nagarajan Pattabiraman, Vincent C. O. Njar, John D. Imig, Liang Wang, Marja T. Nevalainen

**Affiliations:** 1 Department of Pathology, Medical College of Wisconsin Cancer Center, Medical College of Wisconsin, Milwaukee, WI 53226, USA; cmaranto@mcw.edu (C.M.); vudhane@mcw.edu (V.U.); lsabharwal@mcw.edu (L.S.); 2 Department of Pharmacology and Toxicology, Medical College of Wisconsin Cancer Center, Medical College of Wisconsin, Milwaukee, WI 53226, USA; rverma@mcw.edu (R.V.); jdimig@mcw.edu (J.D.I.); 3 Prostate Cancer Center of Excellence at Medical College of Wisconsin Cancer Center, Medical College of Wisconsin, Milwaukee, WI 53226, USA; plaviole@mcw.edu; 4 Department of Tumor Biology, H. Lee Moffitt Cancer Center, Tampa, FL 33612, USA; jing.jia@moffitt.org (J.J.); liang.wang@moffitt.org (L.W.); 5 Institute of Biochemistry and Molecular Biology, Aachen University, 52066 Aachen, Germany; mueller-newen@rwth-aachen.de; 6 Department of Radiology, Medical College of Wisconsin Cancer Center, Medical College of Wisconsin, Milwaukee, WI 53226, USA; 7 Department of Biochemistry, Medical College of Wisconsin, Milwaukee, WI 53226, USA; mpereckas@mcw.edu; 8 Department of Microbiology and Immunology, and Biomedical Engineering, Medical College of Wisconsin, Milwaukee, WI 53226, USA; sterhune@mcw.edu; 9 MolBox LLC, Silver Spring, MD 20910, USA; np47@georgetown.edu; 10 Department of Pharmacology, Marlene and Stewart Greenebaum Comprehensive Cancer Center, Baltimore, MD 21201, USA; vnjar@som.umaryland.edu

**Keywords:** Stat5, IST5-002, transcriptome, toxicity, cell signaling, small-molecule inhibitors, prostate cancer, toxicity

## Abstract

**Simple Summary:**

There is an unmet medical need for new and potent pharmacological inhibitor drugs for the protein Stat5 that would be orally bioavailable for treatment of several different cancers. Previous work has established a critical role for Stat5 in molecular and clinical progression of prostate cancer to metastatic disease and in the pathogenesis of several leukemias and blood-based disorders. Our group has developed a potent pharmacological inhibitor for Stat5, IST5-002, which targets two critical steps in the activation process of Stat5 in cancer cells. In the present work, we evaluated the characteristics of IST5-002 for further development into a cancer drug. We evaluated whether IST5-002 affects the Stat5 targets genes in prostate cancer, defined more closely its mechanisms of action, and investigated its initial toxicity as the basis for further development in order to enable its entrance into clinical testing in patients. Our study supports optimization of IST5-002 compound for oral bioavailability and for clinical development.

**Abstract:**

Stat5 is of significant interest in the search for new therapeutics for prostate cancer (PC) and hematopoietic disorders. We evaluated the transcriptomic specificity of the Stat5a/b inhibitor IST5-002 (IST5) in PC, defined more closely its mechanisms of action, and investigated the in vivo toxicity of IST5 for further optimization for clinical development. The transcriptomic specificity of IST5 vs. genetic Stat5 knockdown was evaluated by RNA-seq analysis, which showed high similarity with the Pearson correlation coefficient ranging from 0.98–0.99. The potency of IST5 vs. its derivative lacking the phosphate group in suppressing Stat5 was evaluated in two separate but complementary assays. The inhibitory activity of IST5 against kinases was investigated in cell-free assays followed by more focused evaluation in a cell-based assay. IST5 has no specific inhibitory activity against 54 kinases, while suppressing Stat5 phosphorylation and subsequent dimerization in PC cells. The phosphate group was not critical for the biological activity of IST5 in cells. The acute, sub-chronic and chronic toxicity studies of IST5 were carried out in mice. IST5 did not cause any significant toxic effects or changes in the blood profiles. The present work supports further optimization of IST5 for oral bioavailability for clinical development for therapies for solid tumors, hematological and myeloproliferative disorders.

## 1. Introduction

Stat5a/b (Stat5) is critical for growth and progression of solid tumors and hematological malignancies, specifically prostate cancer (PC) [1,2,3,4,5,6,7,8,9,10,11,12,13,14,15,16,17,18,19,20,21,22,23,24,25] and Bcr-Abl-driven leukemias [26,27,28,29,30,31,32,33,34,35]. For therapeutic suppression, transcription factors are typically considered suboptimal pharmacological targets because their function relies on protein–protein and protein–DNA interactions that are not easily disrupted by small molecules. Targeting Stat5, however, offers a unique opportunity for development of pharmacological inhibitors because Stat5 serves both as a cytoplasmic signaling molecule and a nuclear transcription factor, and can be suppressed when located in the cytoplasm as a signaling protein [36,37,38,39,40]. Stat5 comprises two highly homologous isoforms Stat5a and Stat5b, which display >90% amino acid identity and are encoded by genes juxtaposed on chromosome 17q21.2 [36]. Stat5 proteins comprise five functional domains: 1) N-terminal domain, 2) coiled-coil domain, 3) DNA-binding domain, 4) Src-homology 2 (SH2)-domain and 5) C-terminal transactivation domain [36]. The SH2-domain mediates receptor-specific recruitment and Stat5a/b dimerization [36].

**Figure 1:**
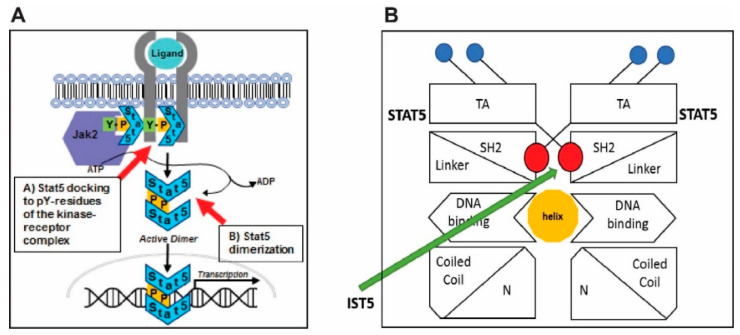
Schematic presentation of the canonical Jak2-Stat5 signaling pathway and the proposed mechanism of action of the Stat5 inhibitor IST5-002 (IST5). (**A**), Stat5 activation is initiated by the binding of an extracellular ligand to a transmembrane receptor. Activation of Stat5 is a two-step process: First, the Stat5 monomer docks transiently to a phospho-tyrosyl moiety of the tyrosine kinase-receptor complex, which results in the phosphorylation of the Y694/699 residue of Stat5 by the tyrosine kinase. Docking of Stat5 to the receptor-tyrosine kinase complex is mediated by the SH2-domain of Stat5 and is, therefore, required for Stat5 phosphorylation. In other words, disruption of the SH2-domain-mediated docking of Stat5 to the receptor-tyrosine kinase complex leads to suppression of Stat5 phosphorylation and activation. Second, the SH2-domain of a phosphorylated Stat5 monomer binds the phosphorylated Y694/699 residue of the partner Stat5 to form a transcriptionally active parallel dimer, which translocates to the nucleus to regulate transcription. In other words, Stat5 dimerization is also mediated by the SH2-domain of Stat5. (**B**), IST5 binds to the SH2 domain of Stat5 monomer and blocks the binding of phosphorylated Stat5 monomer to the pY694/699 residues of the partner Stat5 monomer. In addition, binding of IST5 to the SH2 domain of Stat5 suppresses transient docking of the SH2-domain of Stat5 to the phosphotyrosyl moiety of a tyrosine kinase-receptor complex leading to inhibition of phosphorylation and dimerization of Stat5.

Activation of Stat5 occurs through inducible phosphorylation of a conserved C-terminal tyrosine residue. Activation of Stat5 is a two-step process: First, the Stat5 monomer docks transiently to a phospho-tyrosyl moiety of the tyrosine kinase-receptor complex, which results in the phosphorylation of the Y694/699 residue of Stat5a and Stat5b, respectively, by a tyrosine kinase (Figure 1A) [41,42,43,44,45,46]. Docking of Stat5 to the receptor-tyrosine kinase complex is mediated by the SH2-domain of Stat5 and is required for Stat5 phosphorylation [36,38,41] (Figure 1A,B). Therefore, disruption of the SH2-domain-mediated docking of Stat5 to the receptor-tyrosine kinase complex leads to suppression of Stat5 phosphorylation and activation. Second, the SH2-domain of a phosphorylated Stat5 monomer binds the phosphorylated Y694/699 residue of the partner Stat5 to form a transcriptionally active parallel dimer, which translocates to the nucleus to regulate transcription [36,38,41] (Figure 1A). In other words, also the dimerization of Stat5 dimerization is mediated by the SH2-domain of Stat5.

Using in silico screening, combined with medicinal chemistry, we developed a lead compound Stat5a/b inhibitor, IST5-002 (IST5) [10,24,47]. Our strategy was to target Stat5 as a cytoplasmic signaling molecule by blocking the lower affinity SH2-domain-pY interaction that mediates docking and phosphorylation of Stat5 to the receptor-kinase complex which prevents phosphorylation of Stat5, and/or binding of pY-Stat5 monomer to the pY694/699 residue of the SH2-domain in the partner Stat5 monomer, which mediates dimerization of Stat5 (Figure 1). Both of these steps take place in the cytoplasm (Figure 1). In silico screening of small organic molecules led to identification of IST5 [10]. The strategy we used for identification of IST5 differs from the more conventional approach of cloning and subsequent generation of recombinant Stat5 protein in bacteria followed by screening of chemical libraries for compounds with inhibitory activity against recombinant Stat5 in cell-free assays [31,48,49,50,51,52,53,54]. However, while these strategies often produce and identify Stat5 inhibitors with low IC50 in cell-free assays, they often display poor efficacy against the native Stat5 protein in cell-based assays and perform poorly in actual in vivo tumor growth studies conducted in animals.

By targeting Stat5, IST5 has been shown to have high efficacy in cell-based model systems of cancer [10,24,47]. A substantial body of work supports the concept that Stat5 is critical for PC cell viability in vitro and xenograft tumor growth in vivo [1,2,3,4,5,6,7,8,9,10,11,12,13,14,15,16,17,18,19,20,21,22,23,24,25], and IST5 has been shown to induce extensive apoptotic death of PC cells, block growth of PC xenograft tumors in mice in vivo [2,3,4,6,7,8,10,14,15,47], and induce apoptotic death in patient-derived clinical PCs ex vivo in 3D tumor explant cultures [10,47]. These proof-of-concept data on Stat5 as a therapeutic target protein in PC have been supported by the findings on Stat5 activation in PC predicting early recurrence of the disease and early PC-related death [22,23,25]. Stat5 upregulates the hallmarks of the epithelial-to-mesenchymal transition (EMT) that precedes metastasis in PC, and Stat5 promotes DNA repair in PC increasing tolerance of PC to radiation, which is suppressed by IST5 [4,24]. Recent work shows that the second-generation antiandrogen, enzalutamide, induces a hyperactivated Jak2-Stat5 signaling loop in PC, and that IST5 blocks enzalutamide-resistant PC growth in mice [47]. Specifically, 10/10 PC biopsies from patients treated with enzalutamide (median 6 months) had significantly elevated levels of Stat5 activation [47]. In addition to PC, IST5 has been shown to have remarkable efficacy in chronic myeloid leukemia (CML) cells which is another Stat5-driven cancer [10].

In order to evaluate the potential of IST5 for additional lead optimization as a strategy to enable a strong patent portfolio in view of clinical development, we evaluated the transcriptomic specificity of IST5 vs. genetic knockdown of Stat5 in PC cells. Furthermore, we investigated the efficacy and the mechanisms of action of IST5 in blocking the Jak2-Stat5 signaling cascade. Finally, we evaluated acute, sub-chronic and chronic toxicity profiles of IST5 in vivo in mice. Our results show that IST5 has high transcriptomic specificity in PC cells. While suppressing Stat5 phosphorylation in PC cells with high potency, our data demonstrate that IST5 has minimal inhibitory activity against 54 kinases. Using two different assays of Stat5 dimerization, we demonstrate high efficacy of IST5 in suppressing the dimerization step of Stat5 activation in cells. Our data further show that a chemical modification of IST5 without the phosphate group had similar biological activity to IST5 in PC cells. In toxicity studies in vivo, IST5 did not cause any significant acute, sub-chronic or chronic toxic effects or changes in the blood profiles in mice. In summary, the present work supports further optimization of the small-molecule Stat5 inhibitor IST5 for oral bioavailability for clinical development for therapies for solid tumors, hematological malignancies and myeloproliferative disorders.

## 2. Results

### 2.1. The Transcriptomes Regulated by Genetic Knockdown of Stat5 vs. the Stat5 Inhibitor IST5-002 (IST5) Overlap Extensively

**Figure 2:**
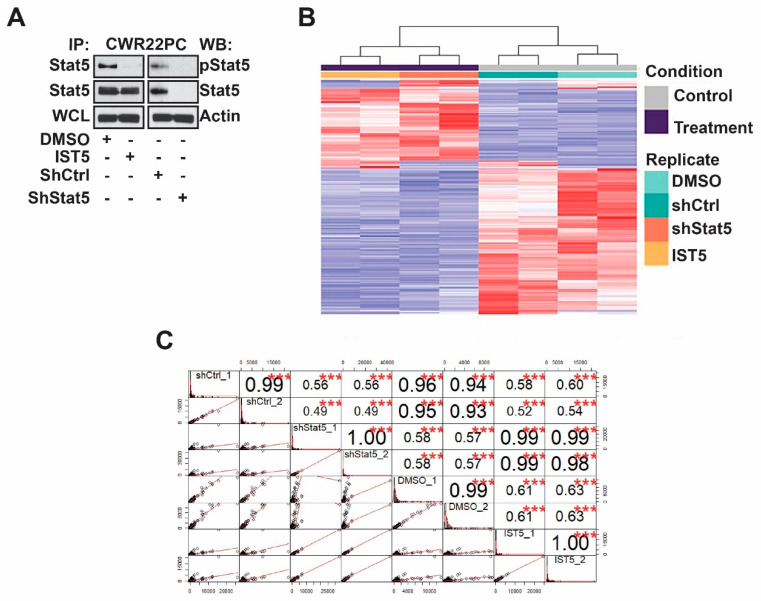
IST5 show high transcriptional specificity in prostate cancer cells. (**A**), Stat5 was suppressed in CWR22Pc cells by lentiviral expression of shStat5 vs. shCtrl or by treatment with IST5 (6 µM) vs. vehicle for 3 days. Stat5 was immunoprecipitated (IP) and immunoblotted (WB) with anti-pYStat5 mAb and anti-Stat5 mAb. Whole-cell lysates (WCL) were immunoblotted with anti-actin pAb as loading control. (**B**), A heatmap demonstrating genes regulated by IST5 vs. genetic knockdown of Stat5 compared to their respective controls (vehicle and shCtrl). Red, higher expression; blue, lower expression. Each column represents a sample and each row represents a transcript. For false discovery rate (FDR) <0.01 and fold change >2 or <−2 as cutoff, 324 and 468 transcripts were upregulated, and 765 and 489 transcripts were downregulated in IST5 and shStat5 treated cells. (**C**), Pearson correlation coefficient ranging from 0.98–0.99 shows a high dependence between the transcriptomes regulated by shStat5 vs. IST5. The distribution histogram of each sample is shown on the diagonal. The bivariate scatter plots with a fitted line are displayed at the bottom and the correlation coefficients are shown on the top of the diagonal. The asterisks indicate the levels of significance associated with the correlations, *** represents the *p* value < 0.001.

In order to evaluate the potential of IST5 for further optimization for clinical development, we compared the IST5-induced transcriptome to that of Stat5 genetic knockdown in PC cells. Stat5 expression was inhibited by lenti-viral expression of shStat5 or shCtrl in CWR22Pc cells for 3 days (Figure 2A). CWR22Pc cells were chosen as the model system because the cell line expresses both Stat5 and androgen receptor and mimics the course of disease progression in PC patient [3,47,55]. When grown in mice as xenograft tumors, CWR22Pc cells form androgen-dependent tumors which regress in response to androgen deprivation but later recur as Castrate-Resistant Prostate Cancer (CRPC) tumors [3,47,55]. In parallel wells, CWR22Pc cells were treated with IST5 or vehicle for 3 days. We performed RNA-seq to profile the gene expression changes and received an average of 22.4 million paired-end clean reads from each sample (Table S5). Using false discovery rate (FDR) <0.01 and fold change >2 or <−2 as cutoff, 324 and 468 transcripts were upregulated in cells in which Stat5 was suppressed by IST5 or shStat5, respectively. At the same time, 765 were significantly down-regulated in cells treated with IST5 vs. 489 transcripts in cells with Stat5 genetic knockdown (Figure 2B). Most importantly, the similarity between the transcriptomes regulated by shStat5 vs. IST5 was remarkably high, with Pearson correlation coefficient ranging from 0.98–0.99 (Figure 2C). 

**Figure 3:**
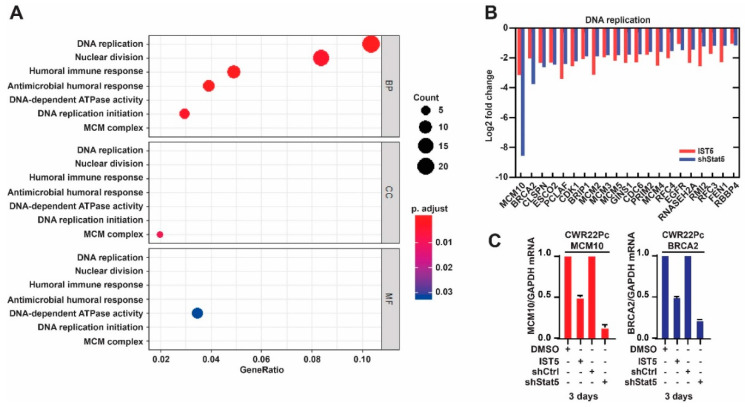
Pathway enrichment analysis of genes regulated similarly by IST vs. genetic knockdown of Stat5 in prostate cancer cells. (**A**), a dot plot of functionally enriched pathways in the genes regulated both by IST5 and genetic knockdown of Stat5 in PC with corresponding adjusted *p* values is shown. The size of each dot represents the number of transcripts in the corresponding pathway and the color indicates the significance. (**B**), a key cluster of 21 genes associated with the enriched pathways related to DNA replication which were regulated by both IST5 and genetic knockdown of Stat5 in PC cells. Log2 fold change is shown. (**C**), mRNA levels of MCM10 and BRCA2 were reduced, determined by quantitative real-time polymerase chain reaction (qRT-PCR), in CWR22Pc cells upon genetic knockdown of Stat5 by lentiviral expression of shStat5 vs. shCtrl or by treatment with IST5 (6 µM) vs. vehicle for 3 days.

Next, we evaluated the most significantly enriched pathways in the transcriptomes regulated by both IST5 and genetic knockdown of Stat5 in PC cells (Figure 3A). The interaction networks among the enriched pathways and the enriched genes are shown in Figure S1. The most significantly enriched pathways regulated by both IST5 and genetic knockdown of Stat5 in PC cells were associated with DNA replication and nuclear division (Figure 3A). Specifically, significant changes were evident in 21 of 203 genes associated with DNA replication and nuclear division (Figure 3B). Specifically, genes associated with minichromosome maintenance family (MCM2, MCM3, MCM4, MCM5, MCM10) were regulated by both IST5 and shStat5 in PC cells, and were associated with DNA replication and DNA replication initiation process as indicated by the functional module analysis (Figure 3). In addition, suppression of the top genes regulated by IST5 and genetic knockdown of Stat5 was confirmed by quantitative real-time polymerase chain reaction (qRT-PCR) in CWR22Pc cells after 3 days of Stat5 inhibition (Figure 3C). It is important to note that the gene expression changes induced by both shStat5 and IST5 in PC cells described here represent later changes in gene expression networks and the early genes (at 24 h) regulated by shStat5/IST5 might be different. In summary, the RNA-seq analysis demonstrated high specificity of the IST5 as the Stat5 inhibitor and revealed involvement of the transcriptome regulated by both IST5 and genetic knockdown of Stat5 in DNA replication processes in PC cells.

### 2.2. IST5 and a Modified IST5 Have Similar Efficacies in Inhibiting Phosphorylation, Dimerization and Transcriptional Activity of Stat5

**Figure 4:**
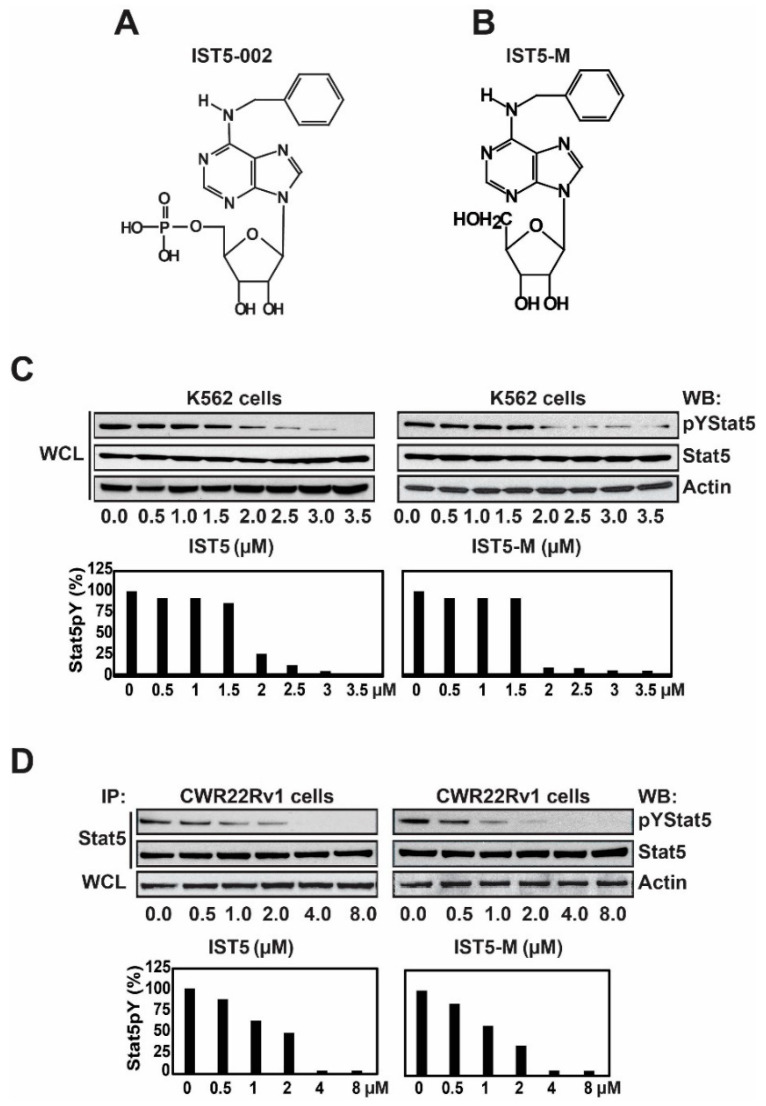
IST5 and the IST5-derivative lacking the phosphate group (IST5-M), both, inhibit Stat5 phosphorylation in CML and prostate cancer cell line with high efficacy. (**A**), chemical structure of IST5-002 (IST5). (**B**), chemical structure of IST5-M (IST5 without the phosphate group. (**C**), IST5 and IST5-M both inhibit Stat5 phosphorylation in CML cells (K562) with high efficacy. K562 cells were treated with IST5 and IST5-M for 4 days at the concentrations indicated. Whole cell lysates (WCL) were immunoblotted with anti-pYStat5 mAb, anti-Stat5 mAb and anti-actin mAb as loading control. (**D**), IST5 and IST5-M, both, decrease Stat5 phosphorylation in PC cells (CWR22Rv1). CWR22Rv1 cells were treated with increasing concentrations of IST5 and IST5-M for 4 days. Stat5 was immunoprecipitated (IP) followed by immunoblotting (WB) with anti-pYStat5 mAb and anti-Stat5 mAb. Whole cell lysates (WCL) were immunoblotted with anti-actin mAb as loading control.

In order to evaluate whether the phosphate group of IST5 is critical for IST5 activity in cell-based assays, we compared the potency of IST5 (Figure 4A) to that of IST5-M (IST5 without the phosphate group) (Figure 4B) in inhibiting phosphorylation of Stat5 in PC and CML cells. CML (K562) cells and PC (CWR22Rv1) cells were cultured for 4 days with increasing concentrations of IST5 and IST5-M (Figure 4C,D). Both IST5 and IST5-M inhibited Stat5 phosphorylation in K562 cells (IC50~1.1 µM) and CWR22Rv1 cells (IC50~1.3 µM) at similar concentrations, where 2 µM of either compound resulted in approximately 75–90% inhibition of Stat5 phosphorylation in K562 cells and 60–75% inhibition in CWR22Rv1 cells. Collectively, these finding indicate that IST5 and IST5-M have similar efficacies in inhibiting phosphorylation of Stat5 in both CML and PC cells.

**Figure 5:**
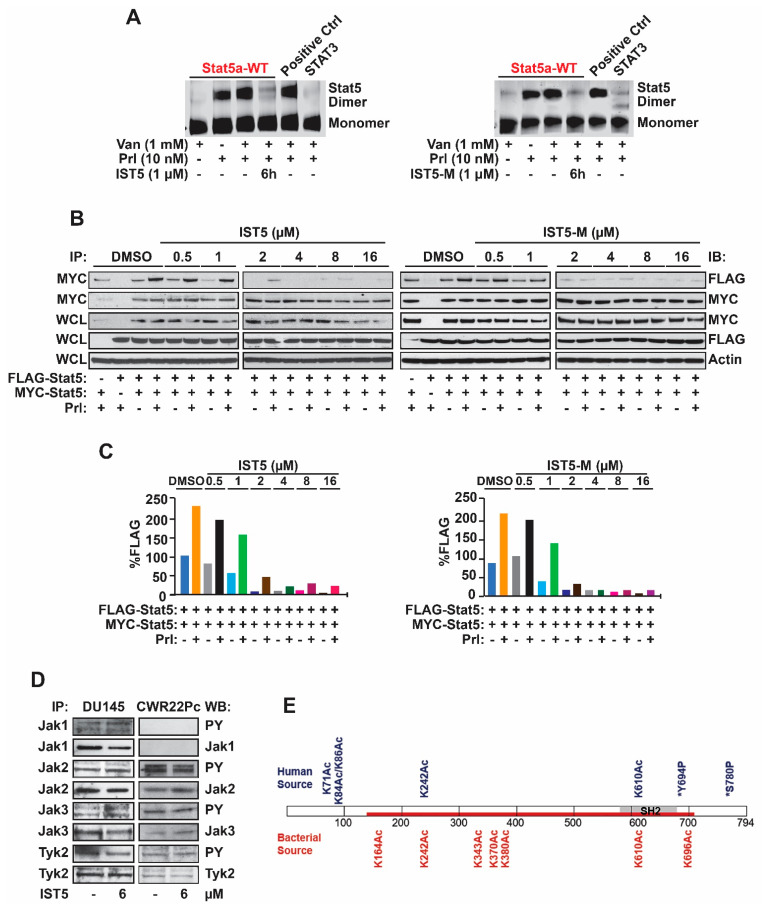
Both IST5 and IST5-M suppress dimerization of Stat5 with similar efficacies in prostate cancer cells. (**A**), IST5 and IST5-M inhibit equally potently dimerization of Stat5 in native-gel-based Stat5 dimerization assay. CWR22Rv1 cells were transfected with STAT5A-eYFP (Stat5 wild type), STAT5A-N642H-eYFP (constitutive activated Stat5 by N642H mutation, as a positive control) or STAT3-eYFP (Stat3 wild type, as a negative control) constructs for 3 days. CWR22Rv1 cells were serum-starved for 16 h, pretreated with vanadate (1 mM) for 2 h, followed by treatment with vehicle, IST5 and IST5-M (1 µM) for 6 h and stimulation with Prl (10 nM) for 1 h. Dimerization of Stat5 was analyzed by detection of the eYFP fluorescence of the native Coomassie gel. (**B**), IST5 and IST5-M inhibited cytokine-induced dimerization of Stat5 with high efficacy in PC cells. PC-3 cells were co-transfected with pCMV-3Flag-Stat5a, pCMV-3Myc-Stat5a, and pPrlR plasmids. Cells were serum-starved for 16 h, pretreated with IST5, IST5-M or vehicle at indicated concentrations for 2 h, followed by stimulation with Prl (10 nM) for 30 min. Anti-MYC mAb was utilized to immunoprecipitate the MYC-tagged Stat5a and blotted with anti-FLAG mAb or anti-MYC mAb, as indicated. Whole cell lysates (WCL) were blotted with anti-MYC mAb, anti-FLAG mAb, or anti-actin mAb to demonstrate the input. (**C**), densitometric analyses of the Stat5 dimerization data based on co-immunoprecipitations and immunoblotting in (**B**). (**D**), IST5 did not alter phosphorylation of Jak1, Jak2, Jak3 or Tyk2 in DU145 and CWR22Pc cells. Prostate cancer cells were treated with IST5 (6 µM) or vehicle for 6 h. Jak1, Jak2, Jak3 and Tyk2 were immunoprecipitated and blotted with anti-phospho-tyrosine, Jak1, Jak2, Jak3 and Tyk2 antibodies. Whole cell lysates (WCL) were immunoblotted for actin. (**E**), differences in posttranslational modifications were detected between native and recombinant STAT5a using mass spectrometry. Native STAT5a from CWR22Rv1 cells and recombinant STAT5a from *E. coli* were gel-purified, digested using trypsin, and analyzed by bottom-up mass spectrometry. Native full-length STAT5a (blue) is show with recombinant STAT5A highlighted (red). Sites of acetylation and phosphorylation from the respective sources are noted including two previously published sites of phosphorylation (asterisk).

Having established that IST5 and IST5-M both interrupt phosphorylation of Stat5, we next investigated the potency of IST5-M vs. IST5 in suppressing dimerization of Stat5 in PC cells. First, we performed a native Polyacrylamide Gel Electrophoresis (PAGE) based Stat5 dimerization assay established originally by Fahrenkamp et al. [56], where CWR22Rv1 cells were transfected with STAT5A-eYFP (Stat5 wild type), STAT5A-N642H-eYFP (constitutive activated Stat5 by N642H mutation, as a positive control) or STAT3-eYFP (Stat3 wild type, as a negative control) constructs for 3 days (Figure 5A). CWR22Rv1 cells were starved for 16 h, pretreated with vanadate (1 mM) for 2 h, followed by treatment with vehicle, IST5 or IST5-M (1 µM) for 6 h and stimulation with Prl (10 nM) for 1 h. As shown in Figure 5A, the treatment of PC cells with IST5 or IST5-M already at 1 µM concentration inhibited robustly Stat5 dimerization in CWR22Rv1 cells. At the same time, the positive control STAT5A-N642H-eYFP showed strong dimer formation, while the negative control STAT3-eYFP failed to dimerize in response to Prl-stimulation, as expected.

To evaluate the efficacies of IST5 vs. IST5-M in suppressing Stat5 dimerization using another assay based on co-immunoprecipitation of MYC-tagged Stat5 with FLAG-tagged Stat5, we generated FLAG- and MYC-tagged Stat5a to transfect PC-3 cells together with a plasmid encoding PrlR (Figure 5B). The cells were serum-starved and treated with IST5 or IST5-M or vehicle before stimulation with Prl (10 nM) for 30 min. Cell lysates were immunoprecipitated with anti-MYC antibody and immunoblotted with anti-FLAG antibody to evaluate the level of dimerization. Immunoblotting of the immunoprecipitates with anti-MYC antibody was conducted to confirm equal transfection levels. Protein input levels were analyzed by immunoblotting of whole cell lysates (WCL) with anti-FLAG, anti-MYC, and anti-actin antibodies, as shown by densitometric analyses of the assay (Figure 5C). Prl stimulation of the cells induced a robust dimerization of Stat5, and this was inhibited equally by both IST5 and IST5-M at IC50 of approximately 1.3 μM (Figure 5C). Collectively, the data indicate that IST5 suppresses both phosphorylation and dimerization of Stat5 in PC cells at approximately similar IC50 of 1.3 μM. Consequently, we propose that the primary mechanism of action of IST5 is disruption of Stat5 docking to the tyrosine-kinase-receptor complex which results in suppression of Stat5 phosphorylation. This is based on the notion that heteromolecular interactions are considerably weaker than interactions between the two partners of homodimers. Moreover, the data presented here demonstrate that both IST5 and IST5-M suppress Stat5 action in cells at equal potency suggesting that the phosphate group of IST5 is not critical for its efficacy in cancer cells.

### 2.3. IST5 Does Not Have Any Significant Inhibitory Activity against a Set of 54 Kinases Including Jak1, Jak2, Jak3 and Tyk3

Given that IST5 suppresses phosphorylation of Stat5 in PC cells, it is possible that IST5 inhibits the kinase(s) responsible for Stat5 phosphorylation and activation in PC cells. To evaluate if IST5 affects kinase activity in cancer cells, we tested kinase inhibitory activity of IST5 in two custom-tailored panels including 54 relevant kinases (Table 1), including Jak1, Jak2, Jak3, Tyk2, Abl1, Abl2, Lck, Fyn, Src and TrkB/C. The data show that IST5 had no significant inhibitory activity against any of the kinases listed in Table 1. In addition, we further tested if IST5 inhibits tyrosine phosphorylation of Jak1, Jak2, Jak3 or Tyk2 using a cell-based assay (Figure 5D), where phosphorylation of the kinase is indicative of enzymatic kinase activity. DU145 and CWR22Rv1 cells were treated with IST5 (6 µM) for 6 h followed by immunoprecipitation of Jak1, Jak2, Jak3 and Tyk2 and immunoblotting with anti-phospho-tyrosine antibody. As shown in Figure 5D, IST5 did not alter phosphorylation of Jak1, Jak2, Jak3 or Tyk2 in neither DU145 cells nor CWR22Pc cells. Collectively, these findings indicate that the inhibitory effect of IST5 on Stat5 phosphorylation in cancer cells is not due to inhibition of Jak2 or other key kinases including Jak1, Jak3, Tyk2, Src, Lck, Fyn, TrkB/C, Abl1 or Abl2 (Table 1, Figure 5D).

### 2.4. Post-Translational Modifications of the Wild-Type Native Stat5 Protein vs. Recombinant Stat5a Protein are Partly Different

Fluorescent polarization assay utilizing recombinant Stat5 has been a common approach for identifying and developing Stat5 inhibitors in the field [31,48,49,50,51,52,53,54]. However, compounds that have high efficacy against recombinant Stat5 in cell-free assays often perform poorly in cell-based assays. For identification of IST5, our approach was to model in silico the SH2 domain of Stat5 for potential pharmacophores that may suppress Stat5 activation in cells and then test the candidate compounds in cell-based assays. Here, we wanted to evaluate whether there are major differences in the post-translational modifications of recombinant Stat5 protein expressed in bacterial cells vs. native WT-Stat5 expressed in human cells. We started an initial comparison of post-translational modifications in the WT-Stat5 vs. recombinant Stat5 using mass spectrometry analysis. CWR22Rv1 cells were infected with lentivirus expressing wild-type Stat5a (WT-Stat5a), and Stat5a was immunoprecipitated from whole cell lysates with purified Stat5a antibody followed by incubation with protein A-Sepharose beads. The native wild-type Stat5a and recombinant Stat5a were separated side by side on 4–12% Sodium Dodecyl Sulfate-Polyacrylamide Gel Electrophoresis (SDS-PAGE)., the proteins bands were excised from the gels and digested with 1 µg of trypsin. In the mass spectrometry analysis, post-translational modifications of native WT-Stat5a vs. recombinant-Stat5a were filtered to include only those identified by two or more unique peptides identified and ranked as high confidence. Database searches with SwissProt databases for *E. coli* and *H. sapiens* showed variable modifications: oxidation, deamidated, methylation, acetylation, dimethylation, phosphorylation, acetylation on N-Terminus, and carbamidomethyl Cys. We observed many of these modifications in both native and recombinant STAT5a (Table S3). The most notable difference in STAT5a was in acetylation with an increase in the number of acetylated sites observed in the recombinant STAT5a as compared to native (Figure 5E, Table S3). Further analysis and studies will be performed on specific post-translational modifications to assess whether these are responsible for the differences in IST5 activity on recombinant Stat5 protein expressed in bacterial cells vs. human cells.

### 2.5. IST5 Does Not Cause Significant Acute or Chronic Toxic Effects in Mice

**Figure 6:**
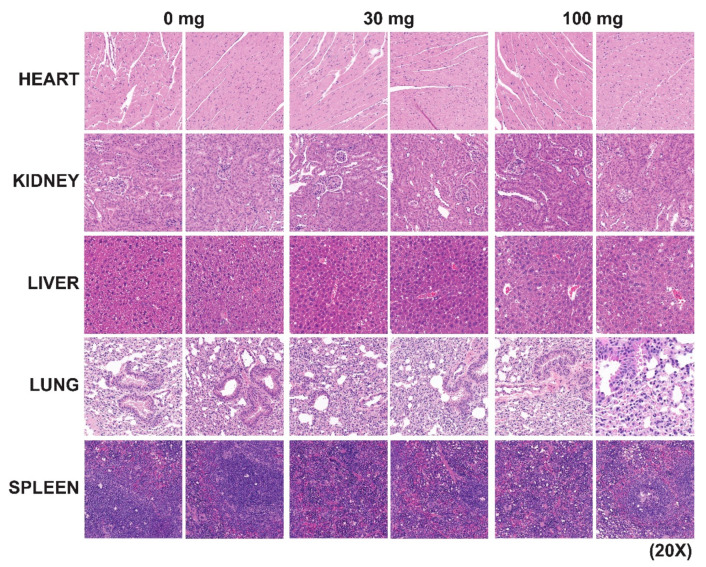
IST5 does show any significant chronic toxicity in vivo in mice. The chronic toxicity studies were carried out in both male and female C57BL/6J and athymic nude mice by daily administration of IST5 vs. vehicle at doses of 0, 10, 30 and 100 mg/kg (*n* = 5/dose). At the end of the study, heart, kidney, liver, lung and spleen tissues were fixed and stained with hematoxylin and eosin. The gross histology of heart, liver, spleen, kidney, spleen and brain were all normal (28 d).

IST5 does not cause any significant acute (Table 2) or chronic (Table 3 and Table 4) toxicity in mice. The acute and chronic toxicity studies were carried out in both male C57BL/6J and athymic nude mice (Table 3 and Table 4) using daily administration of IST5 vs. vehicle at doses of 0, 10, 30 and 100 mg/kg (*n* = 5/dose) as well as in female (data not shown) mice. There were no signs of anemia (hemoglobin-HGB; hematocrit-HCT), thrombocytopenia or leukopenia. In either acute or chronic toxicity studies, there was no mortality in the mice associated with IST5 treatment. The gross histology of the liver, spleen (Figure 6), kidney and brain were normal (28 d). There were no adverse effects on blood clinical chemistry in IST5-treated mice including blood urea, liver enzymes, glucose or albumin, among other parameters (28 d) (Table 3 and Table 4). IST5 had no effects on body weight, organ weight, appearance, respiration, or eye function. Of note, a number of the Jak2 kinase inhibitors in use or in clinical development have shown off-target inhibitory effects on TrkB kinase (a receptor for brain-derived neurotrophic factor), thereby causing neurological side-effects [57,58,59]. IST5 had no inhibitory activity on TrkB (Table 1), and there were no changes in behavior or signs of ataxia, dizziness or confusion in IST5-treated mice (100 mg/kg) or cognitive functions, as assessed by previously described approaches [60,61,62].

## 3. Discussion

A critical role for Stat5 in molecular and clinical progression of prostate cancer (PC) to castrate-resistant and metastatic disease [1,2,3,4,5,6,7,8,9,10,11,12,13,14,15,16,17,18,19,20,21,22,23,24,25] has been established, and Stat5 has been shown to play a key role in pathogenesis of myeloproliferative disorders [26,27,28,29,30,31,32,33,34,35]. However, only a few pharmacological Stat5 inhibitors are available for clinical development. In this work, we show that transcriptomes regulated by IST5 vs. genetic knockdown of Stat5 are markedly similar. While IST5 suppresses Stat5 phosphorylation in PC cells with high potency, our data demonstrated that IST5 has minimal inhibitory activity against 54 most typical kinases present in cancer cells. Using two different assays to evaluate Stat5 dimerization, we demonstrated high efficacy of IST5 in suppressing the dimerization step of Stat5 activation in cells. The phosphate group of IST5, or the lack thereof, did not affect the biological activity of IST5 in cell-based assays. In toxicity studies in vivo, IST5 did not cause any significant acute, sub-chronic or chronic toxic effects or changes in the blood profiles in mice. In summary, the present work supports further optimization of the small-molecule Stat5 inhibitor IST5 for oral bioavailability to enable clinical development of IST5 for therapies for solid tumors, hematological malignancies and myeloproliferative disorders.

One of the key results of this work is the finding of highly redundant gene expression profiles of IST5 vs. genetic knockdown of Stat5 in PC cells. Stat5 expression was inhibited by lenti-viral expression of shStat5 vs. shCtrl and, in parallel wells, CWR22Pc cells were treated with IST5 or a vehicle for 3 days. RNA-seq was performed at three days after suppression of Stat5 by either method to profile the late gene expression changes induced in PC cells by depletion of Stat5 action. The Pearson correlation coefficient of the transcriptomes regulated by IST5 vs. genetic Stat5 knockdown in PC cells ranged from 0.98–0.99 indicating remarkably high dependence. This finding is significant because it suggests that IST5 is a highly specific Stat5 inhibitor in cell-based assays. However, the immediate changes in the gene expression patterns induced by genetic Stat5 knockdown and IST5 in PC cells might be somewhat different from the late changes described here which requires future studies. Future work combining ChIP-Seq and RNA seq analysis of the early transcriptomes regulated by short exposure to IST5 will identify the immediate genes directly controlled by Stat5 binding to their regulatory regions in PC cells. Moreover, single-cell seq analysis of gene expression profiles in preclinical PC tumor models and ex vivo tumor explant cultures of patient-derived PCs will reveal spatial differences, if any, in Stat5-controlled transcriptomes in different cell types present in the tissue architecture of PC.

To evaluate the mechanism of action of IST5 in suppressing Stat5 activity in cancer cells, we demonstrated that IST5 inhibits Stat5 phosphorylation at IC50 values ranging from 1.1–1.3 µM concentrations. Down-regulation of Stat5 phosphorylation by IST5 can theoretically be caused by IST5 blocking Stat5 monomer docking to the activated receptor-Jak2 complex (Figure 1) and/or by IST5 inhibition of Jak2 kinase activity. However, evaluation of the potency of IST5 to inhibit activity of 54 key kinases revealed no significant inhibitory activity of IST5 on any of these kinases. At the same time, IST5 did not suppress phosphorylation of either Jak1, Jak2, Jak3 or Tyk2 in an assay in which the indication of kinase activity is reflected by phosphorylation status of the kinases of the Jak family. To further investigate whether IST5 is able to disrupt dimerization of Stat5 molecules, we employed two separate cell-based assays. In both assays, IST5 suppressed ligand-induced Stat5 dimerization at IC50 of approximately 1.0–1.3 µM concentration. Because both phosphorylation and dimerization of Stat5 were inhibited at similar concentrations of IST5, it is difficult to determine whether disruption of Stat5 dimerization by IST5 is caused by suppression of Stat5 phosphorylation or a direct effect of IST5 in hindering Stat5 dimer-formation or both events occurring at the same time in the cell. However, it is known that heteromolecular interactions are easier to disrupt than interactions between two Stat5 monomers implying that the primary mechanism of action of IST5 may be suppression of Stat5 docking to the receptor-kinase complex. It is important to note that bypassing tyrosine kinases responsible for Stat5 phosphorylation by utilizing direct Stat5 inhibitor would be advantageous for therapy development for Stat5-regulated cancers because targeting Stat5 directly is likely to cause less off-target side effects than targeting kinases or cell surface receptors.

**Figure 7:**
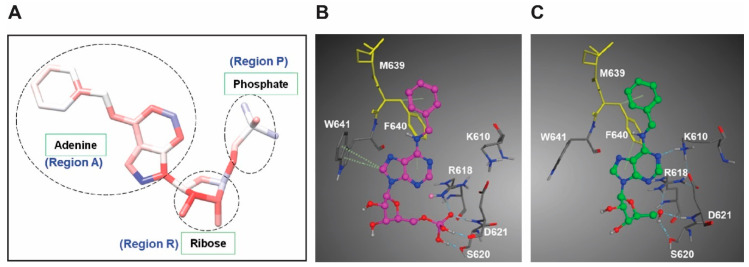
Comparison of the docking of IST5 vs. IST-M to Stat5 in silico. (**A**) Interaction-based color-coded atoms of our inhibitor IST5 and the three regions for modifications. Hydrogens are not shown. (**B**) Comparison of docking of IST5 (1 with the 5′-phosphate group) (**A**) and compound. 2 without 5′-phosphate group (**C**) to Stat5a.

The monophosphate group of IST5, or its exclusion, did not affect the Stat5-inhibitory activity of IST5 significantly in PC cells. This was somewhat surprising since, generally, introduction of phosphate groups into nucleoside analogs make them more polar resulting in decreased transport across the cell membrane. While many phosphates are not soluble in water at standard temperatures and pressure, dihydrogen phosphates are slightly more soluble than the corresponding phosphates and less lipophilic. It is possible that transport proteins for IST5/IST5-M are highly abundant in cancer cells [63]. Based on the identification of the importance of IST5 phosphate group interaction with Stat5b (Figure 7A), we compared the docking interaction of IST and IST5-M (with OH instead of phosphate) the two compounds. As shown in Figure 7B,C, the 5′−OH of IST5-M makes three hydrogen bonds (dashed line) instead of four for the phosphate group in IST5. Due to the loss of phosphate group in IST5-M, the molecule has to move to make the three hydrogen bonds with R618, D621 and S620 (Figure 7C). This leads to the loss of stacking interaction with W641. However, the N3 of adenine forms a hydrogen bond with K610, which is missing in IST5 (Figure 7B). The stacking interactions of the adenine ring with F640 are the same between the two compounds. In summary both compounds bind well to Stat5b but use different kinds of interactions. The comparison of IST5 (with the 5′-phosphate group) and IST5-M (IST5 without the 5′-phosphate group) to Stat5b is shown in Figure 7B,C. Of note, the two amino acid residues that are different in the binding pockets between Stat5a and Stat5b are colored yellow, where M639 and F640 in Stat5b are substituted by N642 and L643, respectively, in Stat5a. In conclusion, the monophosphate group, or the lack thereof, did not significantly affect the biological activity of IST5 in cancer cells. This result is important because it allows the utilization of both IST5 and IST-M to be used as the leads for further optimization for oral other desired formulations.

Transcription factors are typically considered suboptimal targets because their function relies on protein-protein/protein-DNA interactions that are not easily disrupted by small molecules. We developed a lead Stat5 inhibitor, IST5, using in silico screening combined with medicinal chemistry followed by efficacy testing in cell-based assays. More conventional strategies typically involve screening of chemical libraries in cell-free assays against a recombinant Stat5 protein which yields compounds that have high efficacy against recombinant Stat5 but may perform less well in cell-based assays. Our mass-spectrometry analysis-based comparison of post-translational modification in WT-Stat5 vs. recombinant Stat5 showed several potential variations, which warrant more focused future studies.

Our recent work demonstrated sustained Jak2-Stat5 phosphorylation in castrate-resistant PC promoting PC growth during anti-androgen treatment, and high efficacy of IST5 blocking the recurrent PC growth in PC xenograft tumors grown in mice [47]. At the same time, in separate acute and chronic toxicity studies presented here, IST5 caused no mortality or any apparent adverse effects based on functions of the main organ systems (lungs, kidney, spleen, liver, brain), blood profile or neurological functions. Generally, base-modified nucleosides, such as cladribine, clorafabine, fludarabine and gemcitabine, have substantially impacted the treatment of cancer. This work demonstrated that such a base-modified nucleoside IST5 is a potent Stat5 inhibitor with high transcriptomic specificity in targeting Stat5 and low in vivo toxicity providing the rationale for further optimization for oral bioavailability to enable clinical development for treatment of PC and hematological malignancies. Due to often unexpected side-effects becoming evident during the phase II–III clinical development, it is important to note that independent sets of Stat5 inhibitors from multiple different laboratories are needed for successful completion of clinical drug development for a Stat5 inhibitor for therapeutic use in humans.

## 4. Materials and Methods

### 4.1. Cell Lines and Reagents

CWR22Rv1, PC-3, DU145, and CWR22Pc [55] human PC cell lines, and K562 CML-cells (all from America Type Culture Collection), were cultured in RPMI 1640 growth media (Mediatech, Carlsband, CA, USA) containing 10% fetal bovine serum (FBS; Quality Biological) and penicillin/streptomycin (50 IU/mL and 50 µg/mL, respectively; Mediatech, Carlsband, CA, USA). CWR22Pc cells were cultured in the presence of dihydrotestosterone (DHT) (0.8 nM) (Sigma-Aldrich, St. Louis, MO, USA). All cell lines were regularly authenticated by observation of cell morphology, androgen-responsiveness and expression of cell line specific markers and tested for mycoplasma contamination (PCR Mycoplasma Detection Set; Takara Bio Inc., Kusatsu, Shiga, Japan) every 3 months. IST5-002 (IST5) and a modified IST5 (IST5-M) without the phosphate group were both provided by Fox Chase Chemical Diversity Center (Philadelphia, PA, USA). Recombinant human prolactin (Prl) was obtained from NIDDK Hormone and Peptide Program, Torrance, CA.

### 4.2. ShRNA and cDNA Constructs and Lentiviral Production of shRNA

The RNAi Consortium (TRC) pLKO.1 lentiviral vector containing shRNA targeting Stat5a, Stat5b, or scrambled control sequences, were purchased from Open Biosystems, Dharmacon, Lafayette, CO, USA. Wild type Stat5a sequence was cloned into the pLCP plasmid (pLCP-mStat5a-WT) using InFusion Cloning kit (Second-generation, Thermo Fisher Scientific, Waltham, MA). VSV-G pseudotyped high-titer lentiviruses were generated by transient co-transfection of HEK293 cells, with a three-plasmid combination as follows: 9 µg pLKO.1 lentiviral vector containing shRNA of interest, 10 µg pHR’8.2ΔR packaging plasmid and 1 µg pCMV-VSV-G envelope plasmid using lipofectamine 2000 (Life Technologies, Brown Deer, WI, USA) in Opti-MEM (Life Technologies, Brown Deer, WI, USA). CWR22Pc cells were transduced with 200–250 µl of lentiviral shRNA supernatant in the presence of polybrene (1:1000; Sigma-Aldrich) for 3 days to induce >80% knockdown or activation of the protein of interest. STAT5A-eYFP, STAT5A-N642H-eYFP and STAT3-eYFP constructs were provided by Dr. Gerhard Muller-Newen [56] (Institute of Biochemistry and Molecular Biology, RWTH Aachen University, Aachen, Germany).

### 4.3. RNA Extraction and Quantification

Total RNA of each replicate sample was isolated using RNeasy Micro Kit (QIAGEN, Valencia, CA, USA) and reverse transcribed with Super-Script III Reverse Transcriptase (Life Technologies, Brown Deer, WI, USA) following the manufacturer’s instructions. In order to eliminate the potential contaminating DNA, the samples were treated with DNase I (QIAGEN). RNA quality was assessed by Agilent Bioanalyzer 2100 (Agilent, Santa Clara, CA, USA), and RNA integrity number (RIN) ≥7.5 was qualified for subsequent RNA-seq analysis.

### 4.4. Quantitative Real-Time Polymerase Chain Reaction (qRT-PCR)

Total RNA of each replicate sample was isolated using RNeasy Micro Kit (QIAGEN, Valencia, CA, USA) and reverse transcribed using Super-Script III Reverse Transcriptase (Life Technologies, Brown Deer, WI, USA). Quantitative PCR was carried out using MCM-10 (EXON6 F: 5′- GAA GAA GGT TAC GCC ACA GAG -3′ and EXON8 R: 5′- TTT ACA GGT TCC CAG GTC AAG -3′), BRCA-2 (EXON22 F: 5′ CAT ACA GTT AGC AGC GAC AAA AA -3′ and EXON 23 R: 5′ CAA GAT GGC TGA AAG TCT GGAT-3′), Glyceraldehyde 3-phosphate dehydrogenase (GAPDH) (F: 5′-TCA AGA AGG TGG TGA AGC AG-3′ and R: 5′-CTT ACT CCT TGG AGG CAA TG-3′) primers and HotStart-IT SYBR Green One-Step qRT-PCR Master Mix (Affymetrix, Santa Clara, CA, USA). Relative changes in expression levels were determined by a comparative CT method using the formula 2-ΔΔCT; where CT is the threshold cycle of amplification and expressed per the levels of GAPDH mRNA.

### 4.5. RNA-seq Library Preparation and Data Analysis

We performed RNA-seq to evaluate the transcriptomes regulated by IST5 vs. genetic knockdown (lenti-shStat5) of Stat5 with corresponding controls (vehicle or lenti-shCtrl). Total RNA (2 μg) from each sample was used for RNA-seq library preparation after rRNA depletion step. All sequencing libraries were prepared according to the protocol of NEBNext Ultra Ⅱ RNA library preparation kit (NEB, Ipswich, MA, USA) and sequenced on an Illumina HiSeq 2500 platform (Illumina, Inc., San Diego, CA, USA). Sequencing data (fastq files) were quantified with a software tool Salmon (version 1.1.0) [64]. A prebuilt transcriptome index was generated using GENCODE annotations (v32) [65]. Data normalization and differential expression analysis [66] were performed using the DESeq2 R package (version 1.26.0). Transcripts with less than 10 median read-counts were filtered out. We utilized the Benjamini–Hochbert (BH) procedure to adjust for multiple testing. Statistical significance was determined at a FDR threshold <0.01. Performance Analytics R package (version 2.0.4) was applied for visualization of Pearson correlation coefficient. Differentially expressed transcripts were visualized using heatmap R package (version 1.0.12).

### 4.6. Gene Set Enrichment Analyses

To explore the potential molecular pathways involved in the transcriptomes regulated by IST5 vs. genetic knockdown of Stat5, we performed Gene Ontology (GO) analysis using R package clusterProfiler (version 3.14.3) [67]. A gene set that contained genes annotated by the same GO term was defined as functional annotation cluster. The adjusted *p* values (FDR) were estimated to prevent high false positive rate.

### 4.7. Protein Solubilization, Immunoprecipitation and Immunoblotting

Cell pellets were solubilized in the lysis buffer (10 mM Tris-HCl (pH 7.6), 5 mM EDTA, 50 mM sodium chloride, 30 mM sodium pyrophosphate, 50 mM sodium fluoride, 1 mM sodium orthovanadate, 1% Triton X-100, 1 mM phenylmethylsulfonyl fluoride, 5 µg/mL aprotinin, 1 µg/mL pepstatin A and 2 µg/mL leupeptin), and protein concentrations of clarified cell lysates were determined by a simplified Bradford method (Bio-Rad, Hercules, CA, USA). The whole cell lysates were immunoprecipitated with polyclonal antibodies (Table S1) followed by incubation with protein A-Sepharose beads (GE Healthcare, Chicago, IL, USA). The cell lysates and immunoprecipitated proteins were separated on 4–12% SDS-PAGE (Life Technologies, Brown Deer, WI, USA) and transferred electrophoretically to polyvinylidene fluoride membrane (Millipore). For immunoblotting, blocking buffer was tris-buffered saline and tween 20 (TBST, 0.15 M NaCl; 0.1% Tween 20; 50 mM Tris, pH 8.0) with 3% bovine serum albumin (BSA). The immunoreaction with the specific antibodies (Table S1) was detected by horseradish peroxidase-conjugated secondary antibodies followed by enhanced chemiluminescence (GE Healthcare).

### 4.8. Native-Gel-Based Stat5 Dimerization Assay

The efficacy of the Stat5 inhibitors to suppress ligand-induced dimerization of Stat5 molecules was evaluated in CWR22Rv1 cells utilizing fluorescently eYFP-labeled constructs, as described [56]: STAT5A-eYFP (Stat5 wild type), STAT5A-N642H-eYFP (constitutive Stat5 activated by the N642H mutation, as positive control) and STAT3-eYFP (Stat3 wild type, as negative control). CWR22Rv1 cells were starved for 16 h, pretreated with vanadate (1 mM) for 2 h, followed by treatment with vehicle, IST5 or IST5-M (1 µM) for 6 h, and stimulation with Prl (10 nM) for 1 h. The cell lysis was performed under native conditions using the lysis buffer of the NativePAGETM Sample Prep kit supplemented with 2% digitonin (Invitrogen, Brown Deer, WI, USA). The lysates were cleared by centrifugation and incubated with Coomassie brilliant blue G-250 and separated for 3 h at 4 °C using the NativePAGETM Bis-Tris gel system with a gradient polyacrylamide gel (4–16%) according to the manufacturer’s instructions. Dimerization of a STAT5A-eYFP construct based on difference in molecular weight of the Stat5 monomer vs. the Stat5 dimer, was analyzed by detection of the eYFP fluorescence with iBright FL1000 system (Thermo Fisher Scientific, Waltham, MA, USA) by excitation with a 488 nm laser line. The emission was detected using a 515–555 nm bandpass filter.

### 4.9. Coimmunoprecipitation-Based Stat5 Dimerization Assay

FLAG-tagged Stat5a and MYC-tagged Stat5a constructs were generated for the analysis of Stat5 dimer formation. Full-length Stat5a was amplified by PCR and subcloned to pCMV-3FLAG and pCMV-3MYC vectors (Genomics, Santa Clara, CA, USA) with EcoR1 and SalI sites, and both constructs were sequenced. PC-3 cells were serum-starved for 16 h, followed by pretreatment with IST5, IST5-M or vehicle (DMSO) for 2 h at indicated concentrations, and stimulated with Prl (10 nM) in serum-free medium for 30 min. Whole-cell lysates were immunoprecipitated with anti-MYC mAb (2 μg/sample; Santa Cruz Biotechnology, Dallas, TX, USA) and immunoblotted with anti-FLAG mAb (1:1000; Genomics), anti-MYC mAb (1:1000; Santa Cruz Biotechnology, Dallas, TX, USA), and anti-actin pAb (1:4000; Sigma-Aldrich, St. Louis, MO, USA).

### 4.10. Cell-Free Kinase Assay

For Jak1, Jak2, Jak3 and Tyk2, a radiometric filtration binding assay was conducted by Reaction Biologicals (Malvern, PA, USA) (Table 1). This radiometric assay is a cell-free assay [68] which is based on conventional filter-binding assays, directly measuring kinase catalytic activity toward a specific substrate [68]. For evaluation of the activity of the other kinases, the Kinase Seeker Technology was utilized (Luceome Biotechnologies, Tucson, AZ, USA) [69,70,71], which is a luminescence-based cell-free assay. In this assay, the reassembly of luciferase fragments is mediated by the interaction of a kinase with an active site-dependent probe. The competitive displacement of the probe by an inhibitor is measured by a change in luminescence signal. Percent activity of each kinase after exposure to IST5 (1 µM) is presented.

### 4.11. Mass Spectrometry Analysis

For comparison of native wild-type (WT) Stat5a to recombinant Stat5a by mass spectrometry, CWR22Rv1 cells were infected with lentivirus expressing wild-type Stat5a at MOI 5 (WT-Stat5a) for 48 h. Cell pellet was solubilized in the lysis buffer, and protein concentration of clarified cell lysate was determined by simplified Bradford method (Bio-Rad, Hercules, CA, USA). The whole cell lysate was immunoprecipitated with purified Stat5a monoclonal antibody (ST5a-2H2, Invitrogen) followed by incubation with protein A-Sepharose beads (GE Healthcare, Chicago, IL, USA). Recombinant Stat5a cDNA (a gift from Dr. Thorsten Berg) [49] was translated to Stat5a protein in *E. coli* by standard methods (Protein Foundry). The immunoprecipitated native WT-Stat5a expressed in mammalian cells and 15 µg of recombinant Stat5a were separated side by side on 4-12% SDS-PAGE (Life Technologies, Brown Deer, WI, USA).

The excised gels bands were washed with water, de-stained with 50% methanol (100 mM) ammonium bicarbonate and washed with ammonium bicarbonate (50 mM). Proteins were reduced with Dithiothreitol (DTT) (10 mM) for 30 min at 37 °C and alkylated with iodoacetamide (55 mM) for 45 min at 37 °C. The bands were washed with ammonium bicarbonate (50 mM), dehydrated twice with 50% acetonitrile in ammonium bicarbonate (50 mM), dried under vacuum, and digested overnight at 37 °C with 1 µg of trypsin in ammonium bicarbonate (50 mM). Peptides were extracted successively with 0.1% Trifluoroacetic acid (TFA) in water, 0.1% TFA in 70% acetonitrile, and 0.1% TFA in 90% acetonitrile, followed by vacuum drying. Samples were cleaned using carboxylate coated paramagnetic particles, and each digest was reconstituted in 60 µL of 2% acetonitrile with 0.1% formic acid.

The samples were analyzed on a Thermo Scientific Orbitrap Fusion Lumos MS (mass spectrometer) via two technical replicate injections using a data-dependent acquisition (DDA) HCD MS2 instrument method as outlined Table S2). Each analysis included 10 µL of the sample. MS data (Table S3) were analyzed using Proteome Discoverer 2.4 (Thermo) platform as outlined (Table S4). Protein identifications were filtered to include only proteins identified by two or more unique peptides and ranked as high confidence.

### 4.12. Acute Toxicity Study of IST5

The acute toxicity study of the compound IST5 was carried out in athymic nude mice (8 weeks) (Crl:NU/NCr-Foxn1n) of either sex (*n* = 8/dose group, 4 of each sex). IST5 compound was administered once (0, 10, 30, and 100 mg/kg) via single intra-peritoneal (i.p.) injection, and the mice in the vehicle (0 mg/kg) group received each a single i.p. injection of 0.3% hydroxypropyl cellulose. Plasma samples were analyzed for liver (AST, ALP, alkaline phosphatase, total bilirubin, bile acid) and kidney functions (BUN, albumin, globulin and albumin-globulin ratio) (Marshfields Labs, WI, USA).

The following clinical chemistry parameters were measured: Aspartate aminotransferase (AST), alanine aminotransferase (ALT), sorbitol dehydrogenase (SDH), alkaline phosphatase (ALP), γ-glutamyltransferase (GGT), lactate dehydrogenase (LDH), bilirubin (TBIL), protein (TP), glucose (GLU), blood urea nitrogen (BUN), creatinine (CREAT), and electrolytes (Na, K, Cl, Ca, CO2).

### 4.13. Chronic Toxicity Study of IST5

Forty athymic nude mice (8 weeks) (Crl:NU/NCr-Foxn1n) of either sex (20 males and 20 females) were randomized into four groups (*n* = 5/group) for either sex. The randomization of the mice was carried out based on their baseline body weight measured on day 0. IST5 or vehicle (0.3% hydroxypropyl cellulose, (Sigma-Aldrich, MO, St. Louis, MO, USA) were administered to the mice by i.p. injection for 28 days. The mice were observed daily for behavior, and body weights were measured on every 3rd day throughout the 28-day experimental protocol. The monitoring was started immediately after the compound administration and continued for 2 h for any acute signs of toxicity. In addition, the mice were monitored daily for any signs of toxicity using several clinical parameters. The clinical signs that were taken into consideration were as follows: 1) weight loss: rapid weight loss (10 g in 2 days) or, weight loss of extended period time (20 g over 4 days); 2) rapid growth of mass or masses; 3) paralysis or paresis (partial paralysis); 4) frank bleeding from any orifice; 5) lesions interfering with eating or drinking; 6) diarrhea (non-formed/soft stool or diarrhea liquid stool); 6) loss of body temperature; 7) appearance: rough coat, porphyrin staining (red or brown staining around eyes, nose, or along front paws, distended abdomen, alopecia; 8) eyes: ocular discharge, pale appearance, distinct icterus (jaundice or yellowing of the eyes); 9) respiration: coughing or wheezing, rales or crackling in the lungs, respiration increased or labored; 10) behavioral: hunched posture, huddling in a corner, lethargy, avoiding cagemate(s), head tilt, tremors (head, body, tail), spasticity (stiff muscles), ataxia (loss of voluntary muscle movement), seizures or convulsions, circling, persistent self-induced trauma, chattering or vocalizations, loss of startle response, loss of righting reflex. The clinical chemistry parameters measured were the same as those used for the acute toxicity study.

### 4.14. Sub-Chronic Toxicity Study of IST5

The sub-chronic toxicity study (10 days) was carried out in C57BL/6J mice (8 weeks) (stock 000664) (*n* = 10/dose group 5 of each sex). IST5 was administered daily (0, 10, 30 and 100 mg/kg) via i.p. injection. Vehicle (0 mg/kg) group mice received a single i.p. injection of 0.3% hydroxypropyl cellulose. Plasma samples were analyzed for aspartate aminotransferase (AST), alanine aminotransferase (ALT), sorbitol dehydrogenase (SDH), alkaline phosphatase (ALP), γ-lutamyltransferase (GGT), lactate dehydrogenase (LDH), bilirubin (TBIL), protein (TP), glucose (GLU), blood urea nitrogen (BUN), creatinine (CREAT), and electrolytes (Na, K, Cl, Ca, CO2) (Marshfield Labs, WI, USA). A detailed hematological analysis was carried out on the sub-mandibular blood samples of mice collected at the end of the protocol and analyzed for white blood cells, red blood cells, hemoglobin, lymphocyte counts, monocytes, eosinophils and granulocytes. Brain, liver, heart, kidney and spleen were harvested, weighed and fixed in 10% buffered formalin and processed for histopathological analysis. In histopathological analysis, gross morphology and ultra-structural change were assessed. The clinical chemistry parameters measured were the same as those used for the acute toxicity study.

## 5. Conclusions

There is an unmet medical need for development of potent orally bioavailable pharmacological inhibitors of Stat5a/b (Stat5) for several different neoplasms. Castrate-resistant (CR) PC is one key example of Stat5-driven cancers. We have developed a potent lead compound Stat5 inhibitor, IST5-002 (IST5), through structure-based in-silico screening combined with medicinal chemistry. The transcriptomic specificity of IST5 vs. genetic Stat5 knockdown was evaluated by RNA-seq analysis, which showed high similarity with Pearson correlation coefficient ranging from 0.98–0.99. Mechanistically, IST5 targets the SH2-domain of Stat5 and blocks SH2-domain-mediated docking of Stat5 to the receptor-kinase complex, which leads to suppression of Stat5 phosphorylation by the tyrosine kinase. In addition, SH2-domain of Stat5 mediates dimerization of two Stat5 molecules, which is a process also inhibited by binding of IST5 to the SH2 domain of Stat5. Furthermore, IST5 displayed no significant inhibitory activity against a panel of 54 kinases including Jak1, Jak2, Jak3 and Tyk2. Our work presented here shows that a chemical modification of IST5 without the phosphate group had similar biological activity to IST5 in PC cells. In the toxicity studies, IST5 caused no acute or chronic toxic effects in mice as reflected by blood profile and parameters of liver, kidney, spleen or neurological functions.

In summary, a critical role for Stat5 in molecular and clinical progression of prostate cancer to castrate-resistant and metastatic disease has been established, and Stat5 has been shown to play a key role in pathogenesis of myeloproliferative disorders. However, a major gap in the field is a lack of a portfolio of potent orally active pharmacological Stat5 inhibitors for clinical development. The present work supports further optimization of the small-molecule Stat5 inhibitor IST5 for oral bioavailability for clinical development for therapies for solid tumors, hematological malignancies and myeloproliferative disorders.

## Figures and Tables

**Table 1:** Inhibitory activity of IST5 (1 µM) against a panel of 54 kinases.

Kinase	Activity (%)	Kinase	Activity (%)
JAK2	95.4	AXL	90.4
JAK1	96.2	FYN	95.8
JAK3	100.0	LCK	88.9
TYK2	100.0	IGF1R	100.0
ABL1	88.3	IKKe	84.8
ABL2	88.6	INSR	91.0
TRKB	89.4	ITK	100.0
TRKC	93.4	LYN	90.4
PIM1	93.9	MET	100.0
PIM2	91.8	MLK1	100.0
SRC	92.1	MLK3	93.5
PAK1	98.8	MSK2	90.0
AKT1	100.0	MUSK	100.0
AKT2	94.3	p38g	100.0
AKT3	95.8	PDGFRA	100.0
AURKA	85.3	PDGFRB	78.4
AURKB	96.0	PLK4	85.8
BLK	100.0	PYK2	95.7
CAMKK1	94.2	SLK	92.6
CAMKK2	100.0	SYK	92.1
DDR1	89.3	TBK1	92.8
DDR2	97.4	TIE1	100.0
EPHA1	94.0	TNK2	98.3
FAK	99.0	PYK1	93.4
FGFR2	85.7	VEGFR2	89.1
FLT1	88.4	YES1	100.0
FLT2/3	93.6	CAMK1D	92.0

**Table 2:** Plasma biochemistry. Acute toxicity (athymic nude mice): plasma biochemistry.

	Liver Function					Kidney Function				Other		
Parameters	AST	ALT	Alk.Ptase	TBIL	Bile Acid	Albumin	Globulin	A-G Ratio	BUN	Chol	BW	HCT
Group	U/L	U/L	U/L	mg/dL	µmol/dL	g/dL		g/dL		mg/dL	g	L/L
*Normal range*	*52–560*	*39–96*	*50–96*	*0.0–0.9*	*N/A*	*2.5–4.0*	*1.0–3.0*	*N/A*	*9.0–39*	*mg/dL*	*20–28*	*L/L*
0 mg/kg	370 ± 116	65.4 ± 13.5	47.4 ± 7.10	0.61 ± 0.16	1.98 ± 11.51	3.65 ± 0.35	1.35 ± 0.08	1.35 ± 0.08	29.6 ± 0.74	115 ± 40.7	24.0 ± 1.07	0.44 ± 0.02
10 mg/kg	284 ± 78.7	62.1 ± 11.0	43.17 ± 3.5	0.51 ± 0.10	0.88 ± 0.40	3.25 ± 0.01	1.4 ± 0.01	2.36 ± 0.01	27.67 ± 1.4	67.80 ±5.0	22.8 ± 0.79	0.43 ± 0.02
30 mg/kg	488 ± 168	100 ± 23.9	57.0 ± 6.82	1.16 ± 0.43	3.35 ± 2.87	4.16 ± 0.59	1.4 ± 0.08	2.82 ± 0.52	29.6 ± 1.79	182 ± 57.8	21.9 ± 089	0.43 ± 0.02
100 mg/kg	377 ± 96.7	73.2 ± 15.4	55.8 ± 5.42	0.62 ± 0.09	2.52 ± 1.28	3.32 ± 0.15	1.28 ± 0.10	2.74 ± 0.35	28.6 ± 1.14	89.4 ± 5.91	22.0 ± 0.97	0.44 ± 0.01

Aspartate Aminotransferase (AST); Alanine Aminotransferase (ALT); Alkaline Phosphatase (ALP); Total Bilirubin (TBIL); Albumin-Globulin (A/G) Ratio; Blood Urea Nitrogen (BUN); Cholesterol (Chol); Body Weight (BW); Hematocrit (HCT).

**Table 3:** Plasma biochemistry. Chronic toxicity (athymic nude mice): plasma biochemistry.

	Liver Function					Kidney Function				Other		
Parameters	AST	ALT	Alk.Ptase	TBIL	Bile Acid	Albumin	Globulin	A-G Ratio	BUN	Chol	BW	HCT
Group	U/L	U/L	U/L	mg/dL	µmol/dL	g/dL		g/dL		mg/dL	gm	L/L
*Normal range*	*52–560*	*39–96*	*50–96*	*0.0–0.9*	*N/A*	*2.5–4.0*	*1.0–3.0*	*N/A*	*9.0–39*	*mg/dL*	*20–28*	*L/L*
0 mg/kg	314 ± 56.8	45.6 ± 3.92	55 ± 2.21	0.24 ± 0.02	2.23 ± 0.41	2.73 ± 0.04	1.38 ± 0.04	1.99 ± 0.08	23.4 ± 0.85	61.8 ± 1.87	23.0 ± 0.4	0.44 ± 0.02
10 mg/kg	329.9 ± 30.9	50.4 ± 3.71	51.3 ± 0.72	0.26 ± 0.02	21.0 ± 12.7	2.69 ± 0.05	1.41 ± 0.04	1.93 ± 0.08	20.5 ± 0.82	65.0 ± 2.09	22.5 ± 1.1	0.43 ± 0.02
30 mg/kg	336 ± 57.1	45.2 ± 3.76	53.0 ± 1.93	0.21 ± 0.01	2.39 ± 0.32	2.74 ± 0.07	1.47 ± 0.07	1.90 ± 0.11	25 ± 1.32	70.3 ± 1.38	23.5 ± 0.6	0.45 ± 0.01
100 mg/kg	327 ± 4.03	50.9 ± 3.83	53.3 ± 1.76	0.24v0.02	2.98 ± 0.29	2.74 ± 0.04	1.39 ± 0.03	1.39 ± 0.03	23.0 ± 0.07	75.9 ± 2.06	23.5 ± 0.9	0.43 ± 0.03

Aspartate Aminotransferase (AST); Alanine Aminotransferase (ALT); Alkaline Phosphatase (ALP); Total Bilirubin (TBIL); Albumin-Globulin (A/G) Ratio; Blood Urea Nitrogen (BUN); Cholesterol (Chol); Body Weight (BW); Hematocrit (HCT).

**Table 4:** Plasma biochemistry and Hematology data.

(**A**). Plasma biochemistry. Sub-chronic toxicity (C57BL/6j mice): plasma biochemistry.																				
	**Liver function**									**Kidney function**							**Other**			
**Parameters**	**AST**		**ALT**		**Alk.Ptase**	**TBIL**		**Bile Acid**		**Albumin**		**Globulin**	**A-G Ratio**		**BUN**		**Chol**		**BW**	**HCT**
Group	U/L		U/L		U/L	mg/dL		µmol/dL		g/dL			g/dL				mg/dL		gm	L/L
*Normal range*	*52–560*		*39–96*		*50–96*	*0.0–0.9*		*N/A*		*2.5–4.0*		*1.0–3.0*	*N/A*		*9.0–39*		*mg/dL*		*20–28*	*L/L*
0 mg/kg	314 ± 56.8		45.6 ± 3.92		55 ± 2.21	0.24 ± 0.02		2.23 ± 0.41		2.73 ± 0.04		1.38 ± 0.04	1.99 ± 0.08		23.4 ± 0.85		61.8 ± 1.87		23.0 ± 0.4	0.44 ± 0.02
10 mg/kg	329.9 ± 30.9		50.4 ± 3.71		51.3 ± 0.72	0.26 ± 0.02		21.0 ± 12.7		2.69 ± 0.05		1.41 ± 0.04	1.93 ± 0.08		20.5 ± 0.82		65.0 ± 2.09		22.5 ± 1.1	0.43 ± 0.02
30 mg/kg	336 ± 57.1		45.2 ± 3.76		53.0 ± 1.93	0.21 ± 0.01		2.39 ± 0.32		2.74 ± 0.07		1.47 ± 0.07	1.90 ± 0.11		25 ± 1.32		70.3 ± 1.38		23.5 ± 0.6	0.45 ± 0.01
Aspartate Aminotransferase (AST); Alanine Aminotransferase (ALT); Alkaline Phosphatase (ALP); Total Bilirubin (TBIL); Albumin-Globulin (A/G) Ratio; Blood Urea Nitrogen (BUN); Cholesterol (Chol); Body Weight (BW); Hematocrit (HCT)																				
(**B**). Hematology data. Sub-chronic toxicity (C57BL/6j mice): plasma hematology.																				
**Parameters**																				
		**WBC**		**RBC**			**HGB**		**PLT**		**LYM**			**MON**		**EOS**		**GRA**		
Group		K/uL		M/uL			g/dL		M/uL		%			%		%		%		
*Normal range*		*3.9–13.96*		*7.14–12.2*			*10.8–19.2*		*565–2159*		*61.2–87.8*			*2.18–11.0*		*0.02–4.51*		*N/A*		
0 mg/kg		4.35 ± 0.85		9.78 ± 1.4			16.0 ± 1.95		543 ± 77.5		62.5 ± 4.85			8.53 ± 1.3		3.59 ± 0.85		28.9 ± 4.45		
10 mg/kg		4.18 ± 0.65		10.59 ± 0.2			19.8 ± 1.4		614 ± 119		67.8 ± 2.0			6.29 ± 0.75		3.88 ± 1.0		25.9 ± 2.3		
30 mg/kg		3.76 ± 0.55		9.61 ± 0.31			18.2 ± 2.5		553 ± 154		63.23 ± 6.7			7.06 ± 0.5		5.29 ± 1.35		29.7 ± 6.7		
White Blood Cells (WBC); Red Blood Cells (RBC); Hemoglobin (HGB); Platelets (PLT); Lymphocytes (LYM); Monocytes (MON); Eosinophils (EOS); Granulocytes (GRA).																				

## Supplementary Materials

The following are available online at https://www.mdpi.com/2072-6694/12/11/3412/s1, Figure S1: Interaction networks among the enriched pathways and the enriched genes. Table S1: Antibodies used in the study. Table S2: Chromatography and mass spectrometry (MS) instrument acquisition settings. Table S3: Stat5a-peptides (upload excel files). Table S4: Mass spectrometry data processing parameters. Table S5: Summary of sequencing data quality.
