# Correction: Maranto et al. Prospects for Clinical Development of Stat5 Inhibitor IST5-002: High Transcriptomic Specificity in Prostate Cancer and Low Toxicity In Vivo. *Cancers* 2020, *12*, 3412

**DOI:** 10.3390/cancers17060932

**Published:** 2025-03-10

**Authors:** Cristina Maranto, Vindhya Udhane, Jing Jia, Ranjit Verma, Gerhard Müller-Newen, Peter S. LaViolette, Michael Pereckas, Lavannya Sabharwal, Scott Terhune, Nagarajan Pattabiraman, Vincent C. O. Njar, John D. Imig, Liang Wang, Marja T. Nevalainen

**Affiliations:** 1 Department of Pathology, Medical College of Wisconsin Cancer Center, Medical College of Wisconsin, Milwaukee, WI 53226, USA; cmaranto@mcw.edu (C.M.); vudhane@mcw.edu (V.U.); lsabharwal@mcw.edu (L.S.); 2 Department of Pharmacology and Toxicology, Medical College of Wisconsin Cancer Center, Medical College of Wisconsin, Milwaukee, WI 53226, USA; rverma@mcw.edu (R.V.); jdimig@mcw.edu (J.D.I.); 3 Prostate Cancer Center of Excellence at Medical College of Wisconsin Cancer Center, Medical College of Wisconsin, Milwaukee, WI 53226, USA; plaviole@mcw.edu; 4 Department of Tumor Biology, H. Lee Moffitt Cancer Center, Tampa, FL 33612, USA; jing.jia@moffitt.org (J.J.); liang.wang@moffitt.org (L.W.); 5 Institute of Biochemistry and Molecular Biology, Aachen University, 52066 Aachen, Germany; mueller-newen@rwth-aachen.de; 6 Department of Radiology, Medical College of Wisconsin Cancer Center, Medical College of Wisconsin, Milwaukee, WI 53226, USA; 7 Department of Biochemistry, Medical College of Wisconsin, Milwaukee, WI 53226, USA; mpereckas@mcw.edu; 8 Department of Microbiology and Immunology, and Biomedical Engineering, Medical College of Wisconsin, Milwaukee, WI 53226, USA; sterhune@mcw.edu; 9 MolBox LLC, Silver Spring, MD 20910, USA; np47@georgetown.edu; 10 Department of Pharmacology, Marlene and Stewart Greenebaum Comprehensive Cancer Center, Baltimore, MD 21201, USA; vnjar@som.umaryland.edu

## Error in Figure

In the original publication [1], there was a mistake in Figure 5D as published. Accidentally the WB of Tyk2PY was inserted twice for both DU145 Jak1PY and CWR22Pc Tyk2PY. However, the correct WBs were presented in the PowerPoint presentation of the uncut WBs presented to the reviewers of the manuscript in the original submission verify that this was honest error in assembling the figure panel of multiple cropped images. The correct cropped image of the Jak1PY for DU145 cells has now been inserted in the revised Figure 5D figure panel.

In the original publication, there was a mistake in Figure 6 as published. In the figure panel of the lung images, the same image for 0 mg was accidentally inserted also as a representative image of lungs of mice treated with IST5 100 mg per day. The correct image has now been inserted to the revised Figure 6.

The authors state that the scientific conclusions are unaffected. This correction was approved by the Academic Editor. The original publication has also been updated.

## Figures and Tables

**Figure 5:**
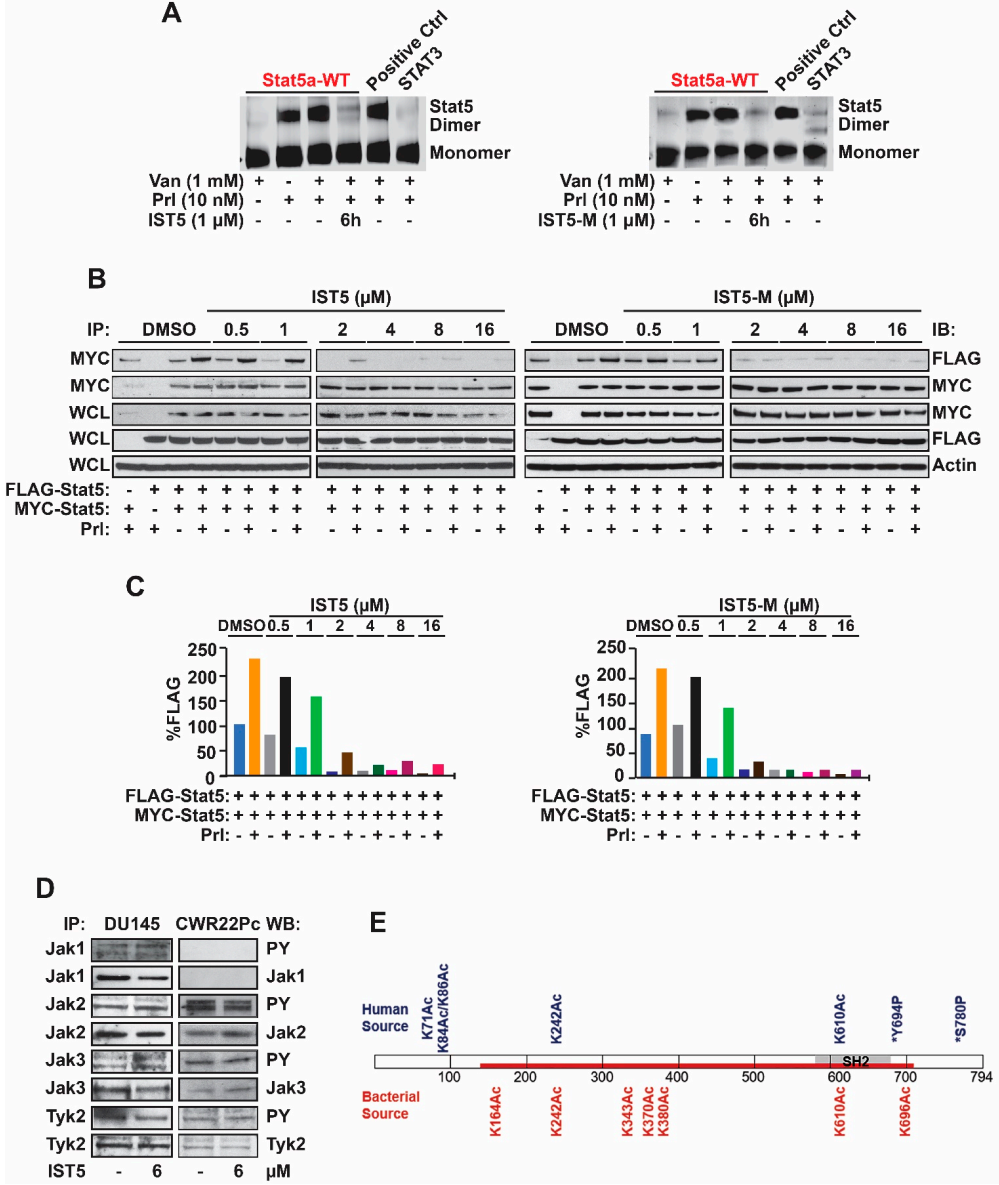
Both IST5 and IST5-M suppress dimerization of Stat5 with similar efficacies in prostate cancer cells. (**A**), IST5 and IST5-M inhibit equally potently dimerization of Stat5 in native-gel-based Stat5 dimerization assay. CWR22Rv1 cells were transfected with STAT5A-eYFP (Stat5 wild type), STAT5A-N642H-eYFP (constitutive activated Stat5 by N642H mutation, as a positive control) or STAT3-eYFP (Stat3 wild type, as a negative control) constructs for 3 days. CWR22Rv1 cells were serum-starved for 16 h, pretreated with vanadate (1 mM) for 2 h, followed by treatment with vehicle, IST5 and IST5-M (1 µM) for 6 h and stimulation with Prl (10 nM) for 1 h. Dimerization of Stat5 was analyzed by detection of the eYFP fluorescence of the native Coomassie gel. (**B**), IST5 and IST5-M inhibited cytokine-induced dimerization of Stat5 with high efficacy in PC cells. PC-3 cells were co-transfected with pCMV-3Flag-Stat5a, pCMV-3Myc-Stat5a, and pPrlR plasmids. Cells were serum-starved for 16 h, pretreated with IST5, IST5-M or vehicle at indicated concentrations for 2 h, followed by stimulation with Prl (10 nM) for 30 min. Anti-MYC mAb was utilized to immunoprecipitate the MYC-tagged Stat5a and blotted with anti-FLAG mAb or anti-MYC mAb, as indicated. Whole cell lysates (WCL) were blotted with anti-MYC mAb, anti-FLAG mAb, or anti-actin mAb to demonstrate the input. (**C**), densitometric analyses of the Stat5 dimerization data based on co-immunoprecipitations and immunoblotting in (**B**). (**D**), IST5 did not alter phosphorylation of Jak1, Jak2, Jak3 or Tyk2 in DU145 and CWR22Pc cells. Prostate cancer cells were treated with IST5 (6 µM) or vehicle for 6 h. Jak1, Jak2, Jak3 and Tyk2 were immunoprecipitated and blotted with anti-phospho-tyrosine, Jak1, Jak2, Jak3 and Tyk2 antibodies. Whole cell lysates (WCL) were immunoblotted for actin. (**E**), differences in posttranslational modifications were detected between native and recombinant STAT5a using mass spectrometry. Native STAT5a from CWR22Rv1 cells and recombinant STAT5a from *E. coli* were gel-purified, digested using trypsin, and analyzed by bottom-up mass spectrometry. Native full-length STAT5a (blue) is show with recombinant STAT5A highlighted (red). Sites of acetylation and phosphorylation from the respective sources are noted including two previously published sites of phosphorylation (asterisk).

**Figure 6:**
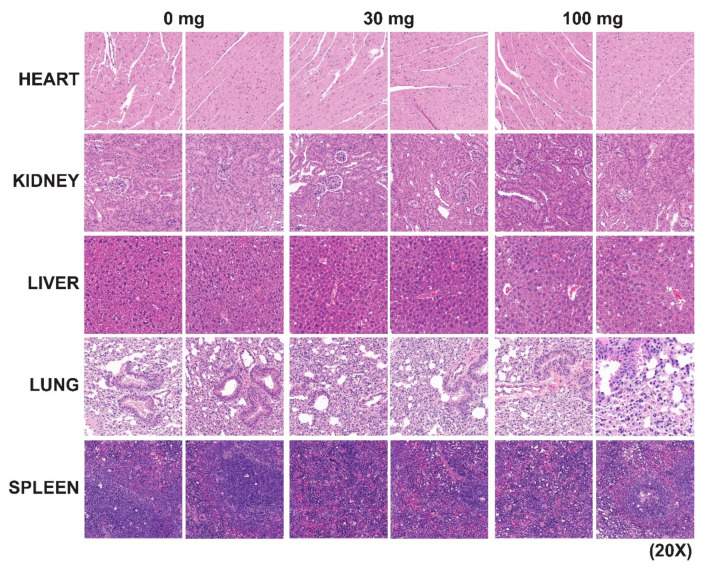
IST5 does show any significant chronic toxicity in vivo in mice. The chronic toxicity studies were carried out in both male and female C57BL/6J and athymic nude mice by daily administration of IST5 vs. vehicle at doses of 0, 10, 30 and 100 mg/kg (*n* = 5/dose). At the end of the study, heart, kidney, liver, lung and spleen tissues were fixed and stained with hematoxylin and eosin. The gross histology of heart, liver, spleen, kidney, spleen and brain were all normal (28 d).
