# Supplementary material for: Prospects for Clinical Development of Stat5 Inhibitor IST5-002: High Transcriptomic Specificity in Prostate Cancer and Low Toxicity In Vivo
Source: Cancers (Basel). 2020 Nov 18;12(11):3412. doi: 10.3390/cancers12113412 (PMC7724566; doi:10.3390/cancers12113412)
Supplement: Supplementary file 1 [file cancers-12-03412-s001.zip › cancers-997294-supplementary/cancers-997294-Supplementary for proofreading.docx]

Supplementary Materials: Prospects for Clinical Development of Stat5 Inhibitor IST5-002: High Transcriptomic Specificity in Prostate Cancer and Low Toxicity In Vivo

Cristina Maranto, Vindhya Udhane, Jia Jing, Ranjit S.Verma, Gerhard Müller-Newen, Peter S. LaViolette, Michael Pereckas, Lavannya Sabharwal, Scott Terhune, Nagarajan Pattabiraman, Vincent C. O. Njar, John D. Imig, Liang Wang and Marja T. Nevalainen


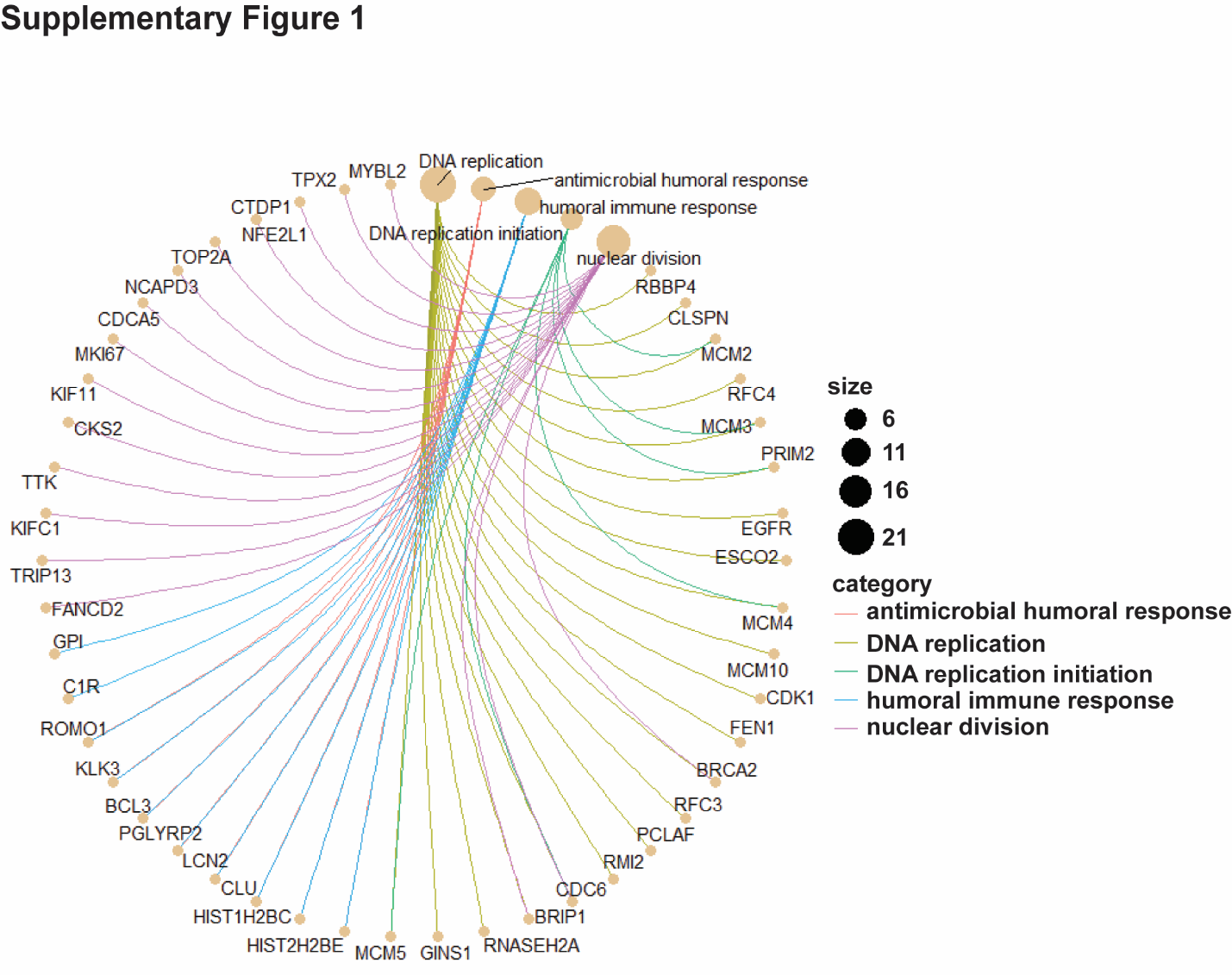


**Figure S1.** Interaction networks among the enriched pathways and the enriched genes. The size of each dot represents the number of transcripts in the corresponding pathway. Different colors represent the pathway categories.

**Table 1.** Antibodies used in the study.

| **Antibody** | **Manufacturer** | **Dilution** | **Application** | **Catalog No.** |  |
| --- | --- | --- | --- | --- | --- |
| Stat5a and Stat5b | Millipore | 4:1000 | IP | Customized | Polyclonal |
| Jak2 | Millipore | 8:1000 | IP | 06-1310 | Polyclonal |
| Jak1 | Cell Signaling | 8:1000 | IP | 3332 | Polyclonal |
| Jak3 | Abcam | 8:1000 | IP | AB45141 | Polyclonal |
| Tyk2 | Millipore | 4:1000 | IP | Customized | Polyclonal |
| pY694 (Stat5) | BD Biosciences | 1:1000 | WB | 611965 | Monoclonal |
| Stat5a/b | BD Biosciences | 1:1000 | WB | 610192 | Monoclonal |
| Jak2 | MyBiosource | 1:1000 | WB | MBS395346 | Monoclonal |
| pYJak2 | Cell Signaling | 1:1000 | WB | 3771S | Monoclonal |
| Actin | Sigma Aldrich | 1:4000 | WB | A2066 | Polyclonal |
| Jak1 | BD Biosciences | 1:1000 | WB | 610232 | Monoclonal |
| Jak3 | Santa Cruz | 1:1000 | WB | sc-6932 | Monoclonal |
| Tyk2 | Santa Cruz | 1:1000 | WB | sc-5271 | Monoclonal |
| PY | Cell Signaling | 1:1000 | WB | 3771S | Monoclonal |
| MYC | Santa Cruz | 1:1000 | WB | sc-40 | Monoclonal |
| FLAG | Genomics | 1:1000 | WB | F3165 | Monoclonal |
| Goat anti-mouse | BD Biosciences | 1:2000 | WB | 554002 |  |
| Goat anti-rabbit | BD Biosciences | 1:2000 | WB | 554021 |  |
| . | | | | |  |

IP, immunoprecipitation; WB, Western blot.

**Table S2.** Chromatography and mass spectrometry (MS) instrument acquisition settings.

| **Sample Volume** | **10 µL** | **Isolation Window** | **1.6 m/z** |
| --- | --- | --- | --- |
| **Stationary Phase** | Thermo Acclaim PepMap C_18_  75 µm × 50 cm | **MS^2^ AGC Target** | 5e4 |
| **LC Solvent A** | 100% H_2_O,  0.1% formic acid | **MS^2^ Maximum IT** | 54 ms |
| **LC Solvent B** | 80% acetonitrile,  0.1% formic acid | **Normalized Collision Energy** | 30 |
| **Gradient Ramp**  **Duration**  **Flow Rate** | 2.5–5% B in 1 min  5–7% B in 4 min  7–28% B in 72 min  28–60% B in 10 min  300 nL/min | **Minimum Intensity Req.** | 50000 |
| **Mass Spectrometer** | Thermo Orbitrap Fusion  Lumos | **Dynamic Exclusion** | 60.0 s |
| **Spray Voltage** | 2.0 kV | **MS^2^ acquisition** | Data dependent, 3 s cycle time,  Centroid |
| **In-Source CID** | 0.0 eV | **MS^2^ Fragmentation** | HCD |
| **MS^1^ scan range** | 375–1500 m/z | **MS^2^ Detection** | Orbitrap |
| **MS^1^ resolution** | 120,000 @ 200 *m/z* | **MS^2^ fixed first mass** | 110 *m/z* |
| **MS^1^ AGC Target** | 4e5 | **MS^2^ resolution** | 30,000 @ 200 *m/z* |
| **MS^1^ Maximum IT** | 50 ms | **Advanced Precursor**  **Determination** | on |

**Table S4.** Mass spectrometry data processing parameters.

| **Platform** | **ProteomeDiscoverer 2.3** | **Target FDR (Strict) for PSMs:** | **0.01** |
| --- | --- | --- | --- |
| **Search Algorithms** | SequestHT | **Target FDR (Relaxed) for PSMs:** | 0.05 |
| **Validation** | Target Decoy PSM Validator  Peptide Validator  Protein FDR Validator | **Target FDR (Strict) for Peptides:** | 0.01 |
| **Database** | SwissProt human created 2019-05-01  SwissProt *E. coli* created 2019-05-01  MaxQuant Contaminants  Recombinant protein sequences as provided | **Target FDR (Relaxed) for Peptides:** | 0.05 |
| **Digest** | Trypsin (Full)  2 Missed Cleavages Allowed |  |  |
| **Precursor mass tolerance** | 10 ppm |  |  |
| **Fragment mass tolerance** | 0.02 Da |  |  |
| **Static Modifications** | Carbamidomethyl (C), |  |  |
| **Dynamic Modifications** | Oxidation (M), acetylation (protein N-terminus), methyl, dimethyl, and acetyl (K) phosphor (S, T, Y), deamidation (N, Q) |  |  |

**Table 5.** Summary of sequencing data quality.

| **Sample** | **Raw reads** | **Clean reads** | **Mappable Rate (%)** | **Q20 (%)** | **Q30 (%)** | **GC Content (%)** |
| --- | --- | --- | --- | --- | --- | --- |
| **shCtrl_1** | 20,405,217.00 | 20,017,556.00 | 98.1 | 97.61 | 93.68 | 50.36 |
| **shCtrl_2** | 21,381,620.00 | 20,902,909.00 | 97.76 | 97.55 | 93.54 | 50.41 |
| **shStat5_1** | 19,981,947.00 | 19,671,686.00 | 98.45 | 97.48 | 93.39 | 51.47 |
| **shStat5_2** | 20,069,903.00 | 19,630,181.00 | 97.81 | 97.78 | 94.11 | 50.84 |
| **DMSO_1** | 19,768,433.00 | 19,276,656.00 | 97.51 | 97.7 | 93.89 | 50.69 |
| **DMSO_2** | 22,293,634.00 | 21,763,900.00 | 97.62 | 97.69 | 93.96 | 50.18 |
| **IST5_1** | 19,941,689.00 | 19,499,777.00 | 97.78 | 97.7 | 93.89 | 49.61 |
| **IST5_2** | 19,640,686.00 | 19,256,927.00 | 98.05 | 97.86 | 94.31 | 50.36 |
