# Supplementary material for: Prospects for Clinical Development of Stat5 Inhibitor IST5-002: High Transcriptomic Specificity in Prostate Cancer and Low Toxicity In Vivo
Source: Cancers (Basel). 2020 Nov 18;12(11):3412. doi: 10.3390/cancers12113412 (PMC7724566; doi:10.3390/cancers12113412)

Figure 2A Left Panel

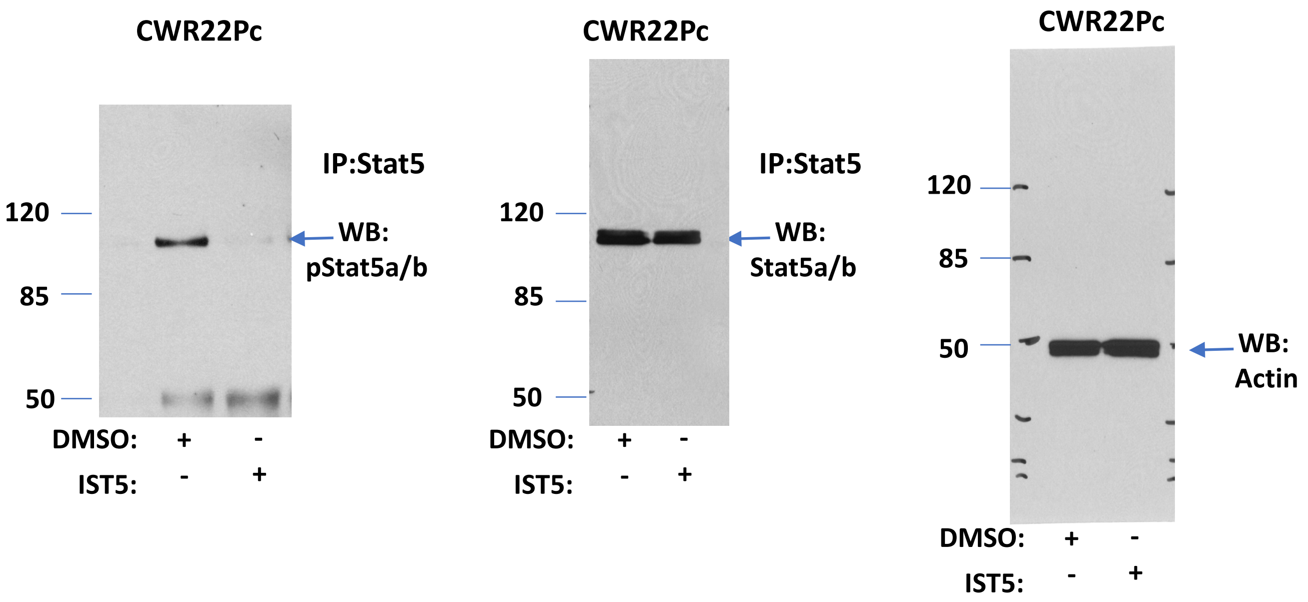

Figure 2A Right Panel

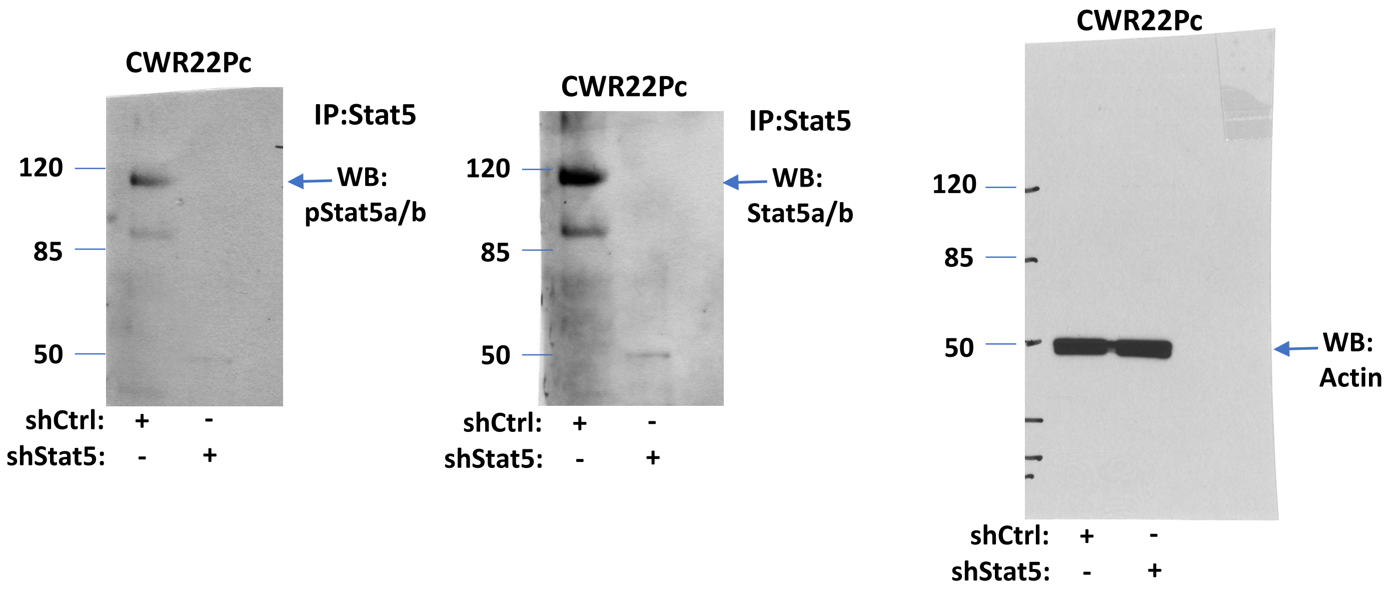

Figure 4C Left Panel

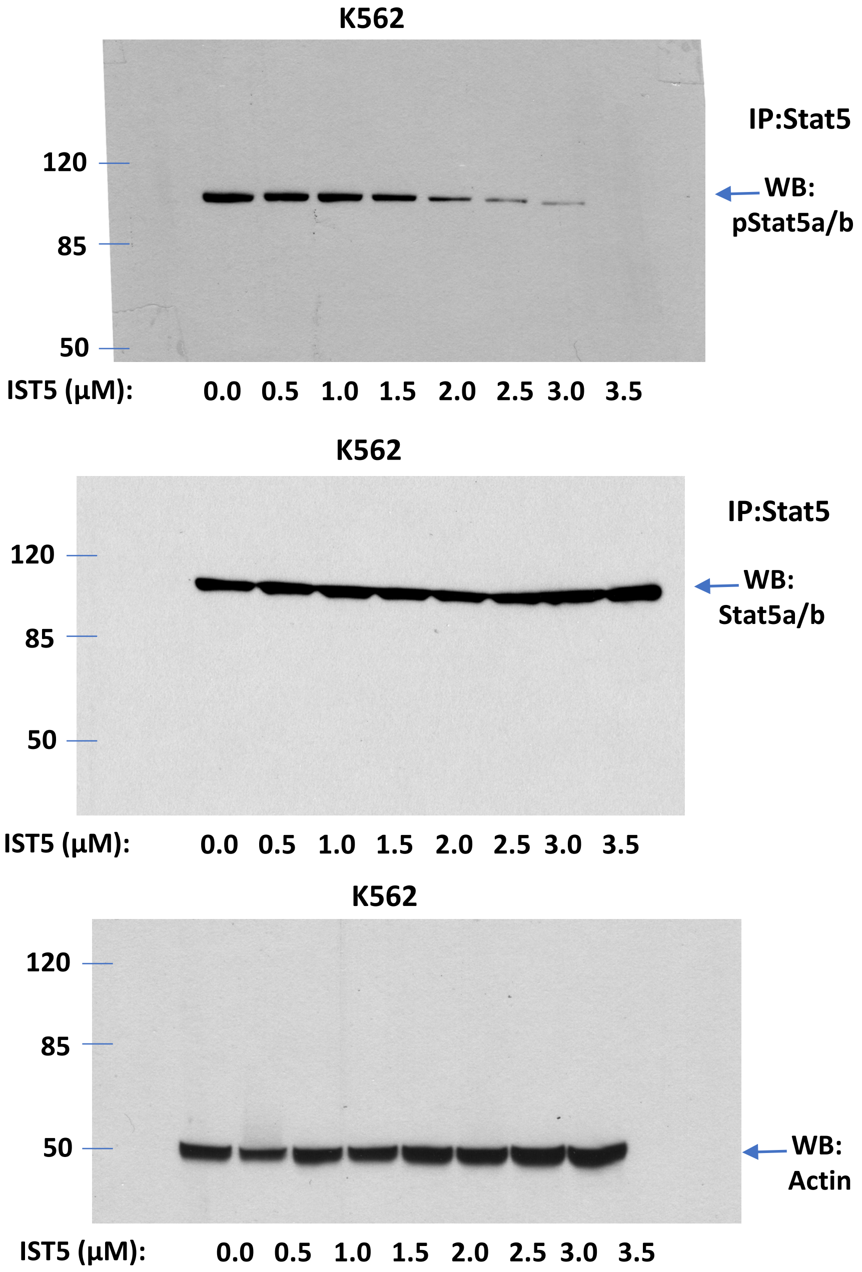

Figure 4C Right Panel

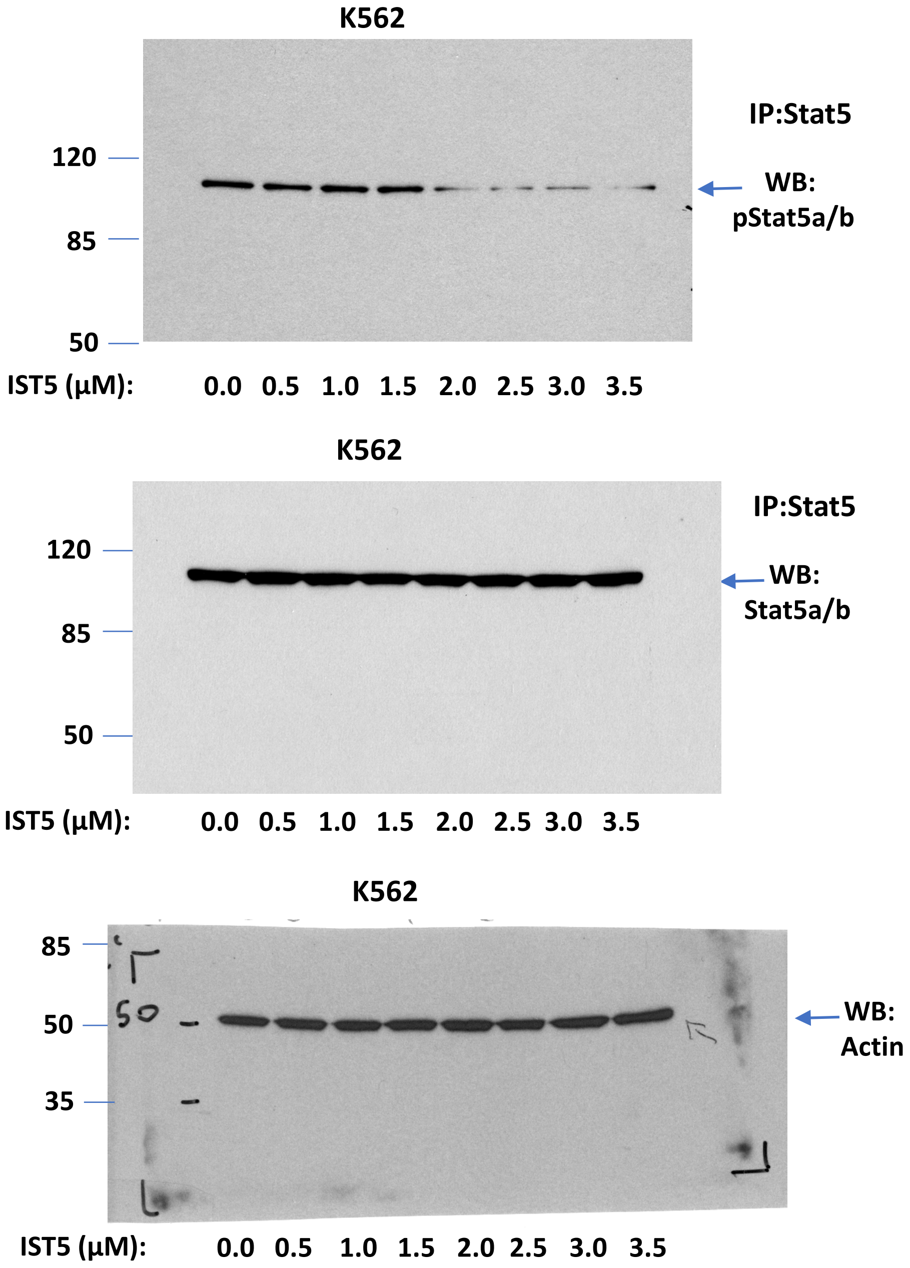

Figure 4D Left Panel

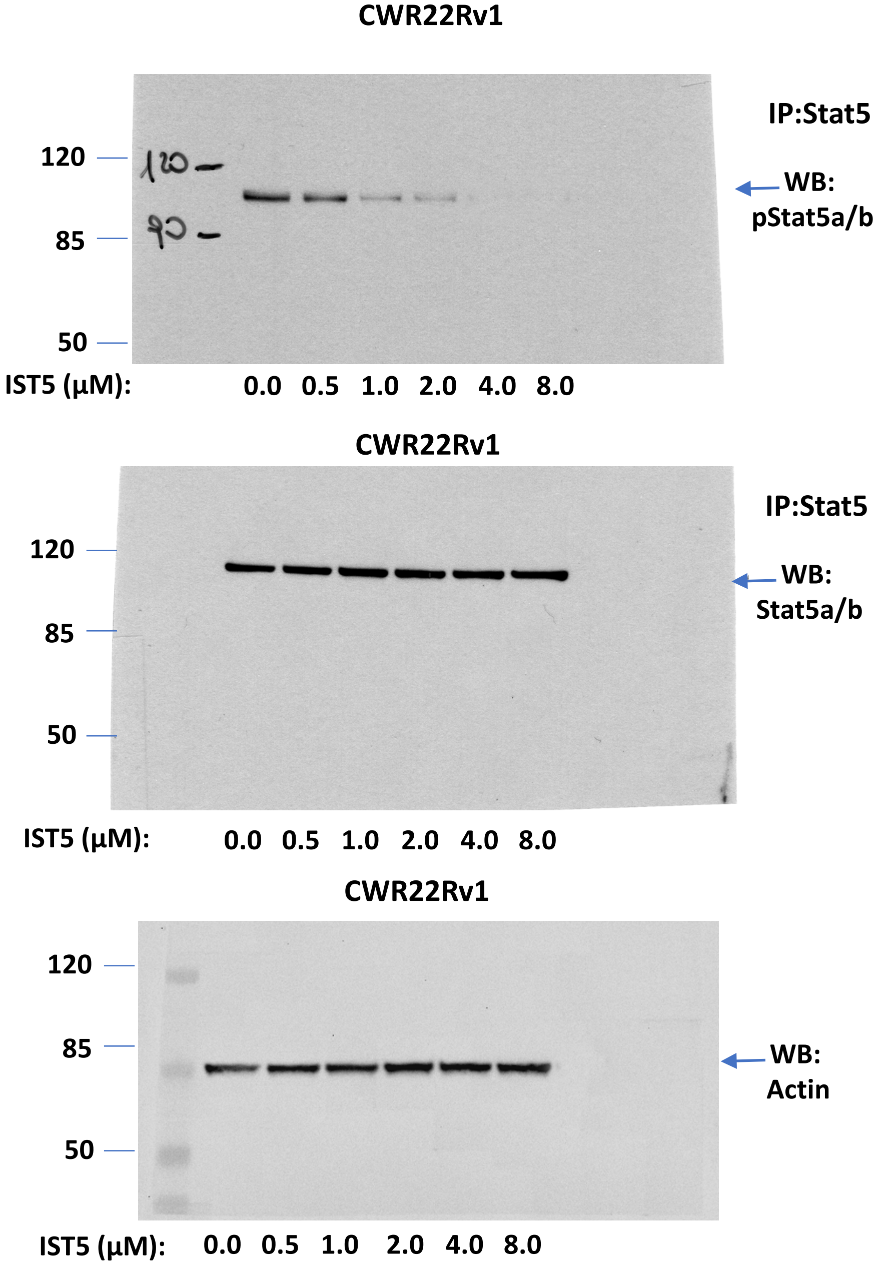

Figure 4D Right Panel

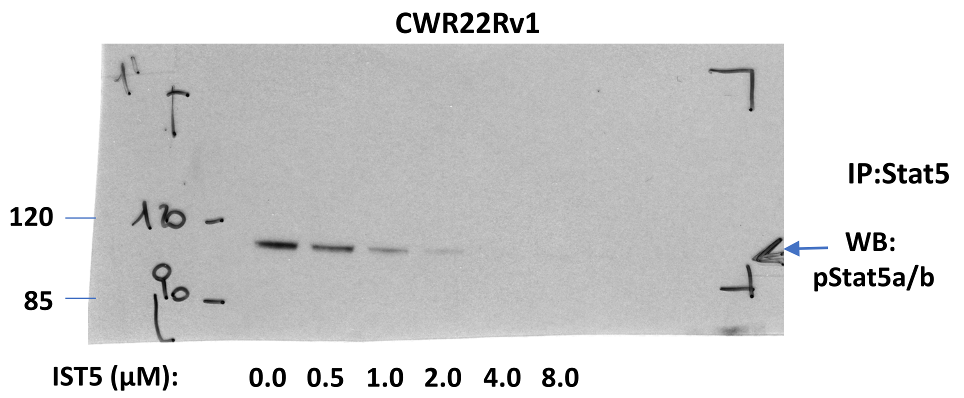

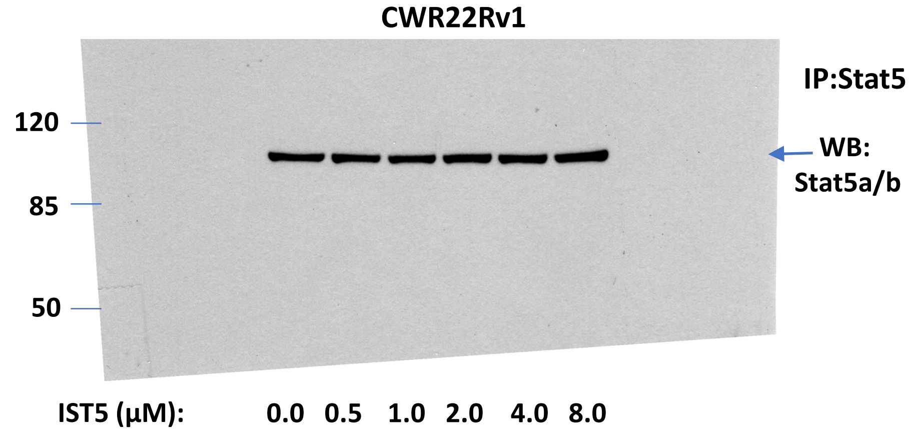

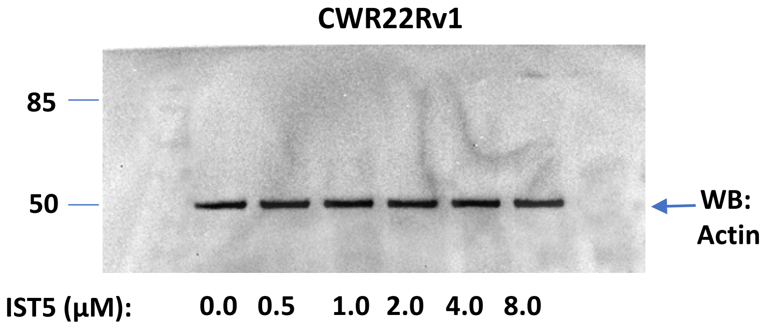

Figure 5B

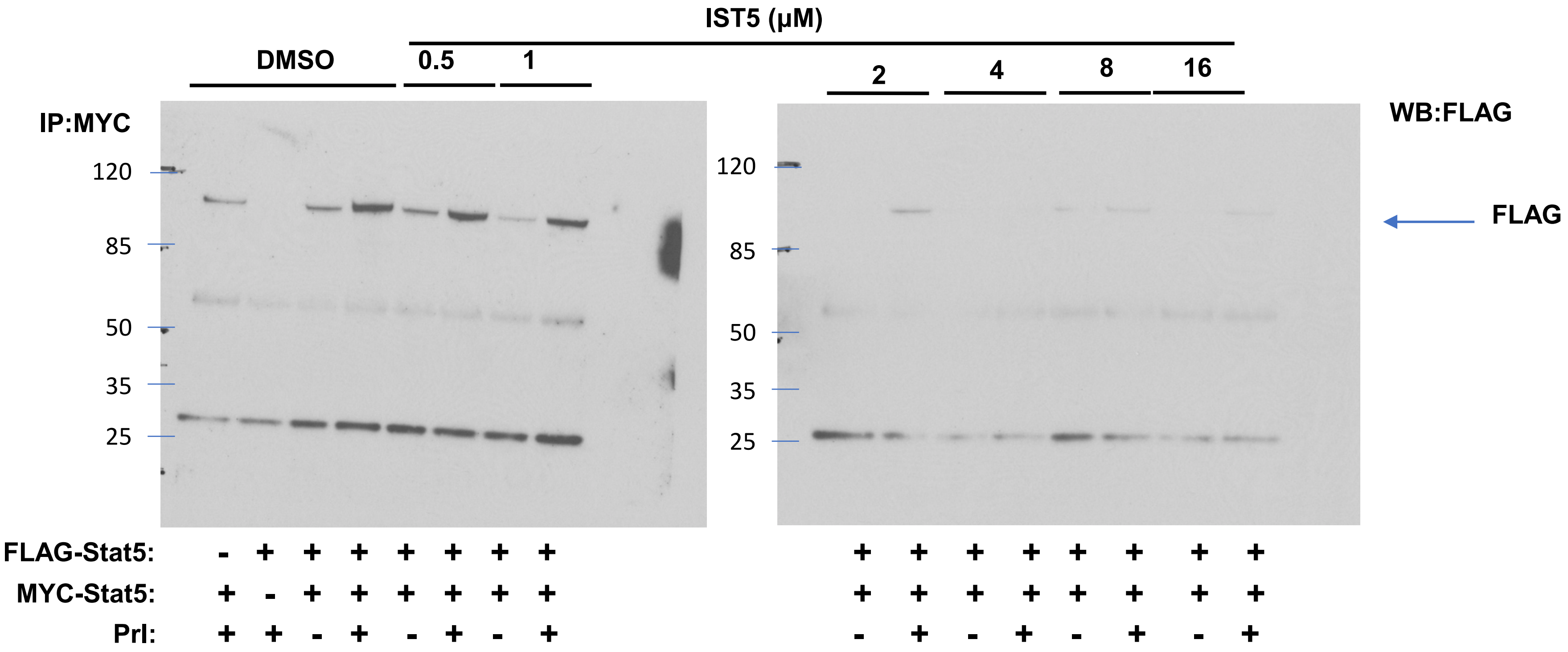
IST5 (µM)
DMSO
0.5
1
2
4
8
16
IP:MYC
WB:FLAG
120
85
50
35
25
FLAG
FLAG-Stat5: - + + + + + + + + + + + + + + +
MYC-Stat5: + - + + + + + + + + + + + + + +
Prl: + + - + - + - + - + - + - + - +

Figure 5B

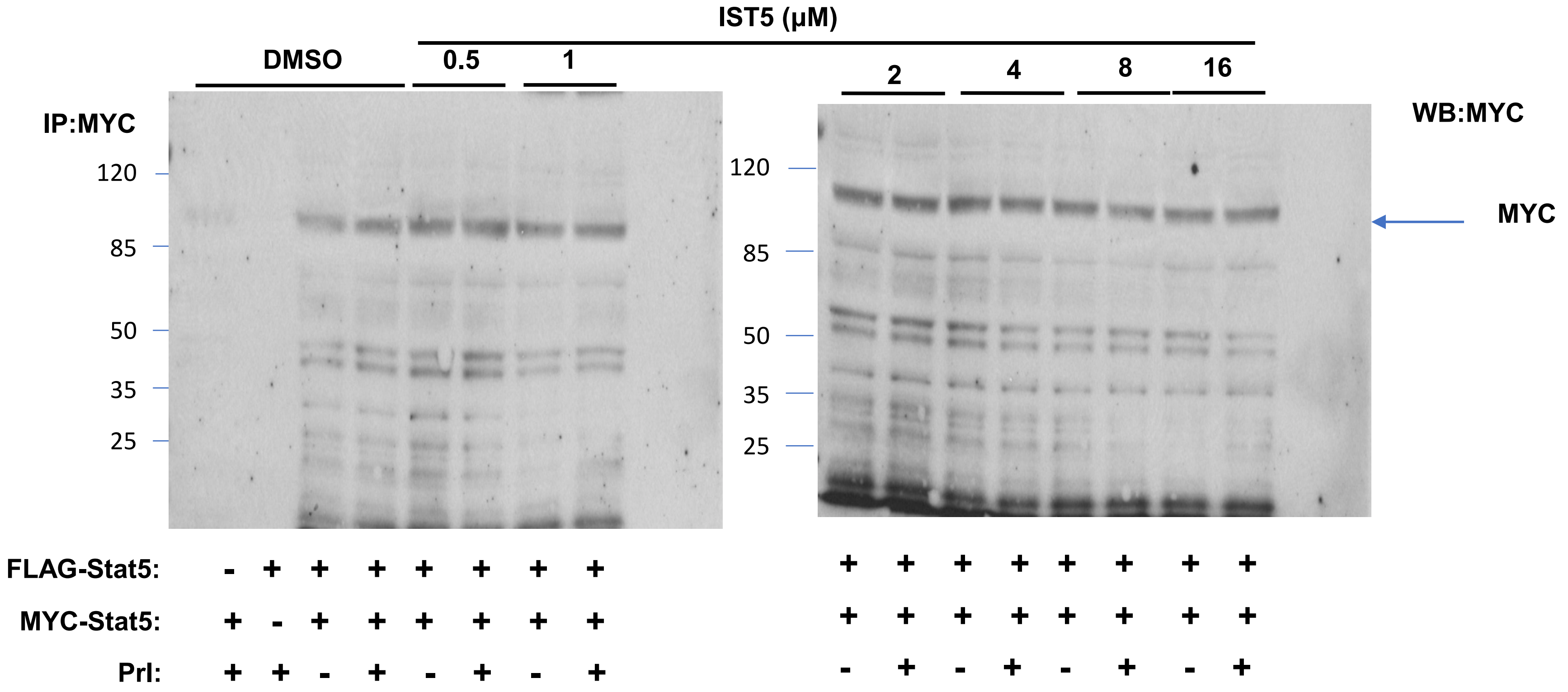
IST5 (µM)
DMSO
0.5
1
2
4
8
16
IP:MYC
WB:MYC
120
85
50
35
25
MYC
FLAG-Stat5:
MYC-Stat5:
Prl:
- + + + + + + +
+ - + + + + + +
+ + - + - + - +
+ + + + + + + +
+ + + + + + + +
- + - + - + - +

Figure 5B

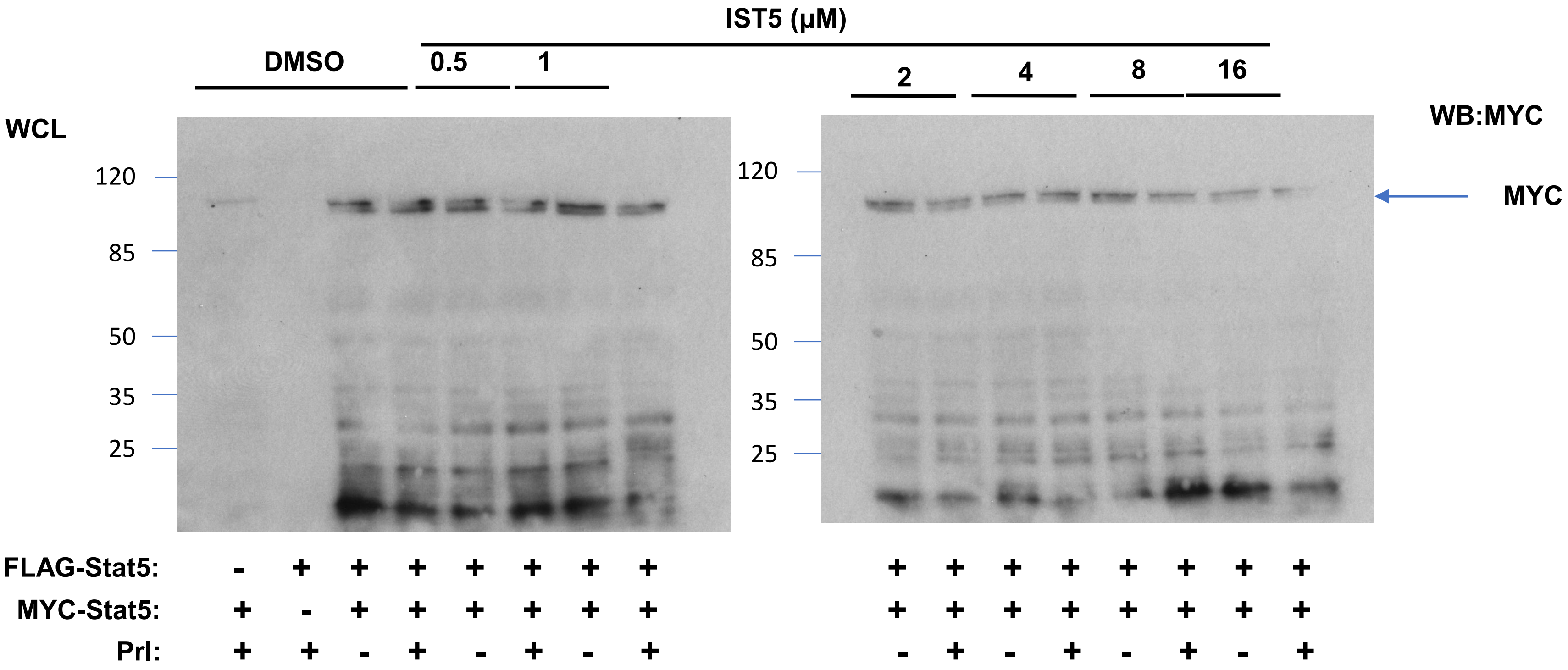
IST5 (μM)
DMSO
0.5
1
2
4
8
16
WCL
WB:MYC
MYC
120
85
50
35
25
FLAG-Stat5: - + + + + + + + + + + + + + + +
MYC-Stat5: + - + + + + + + + + + + + + + +
Prl: + + - + - + - + - + - + - + - +

Figure 5B

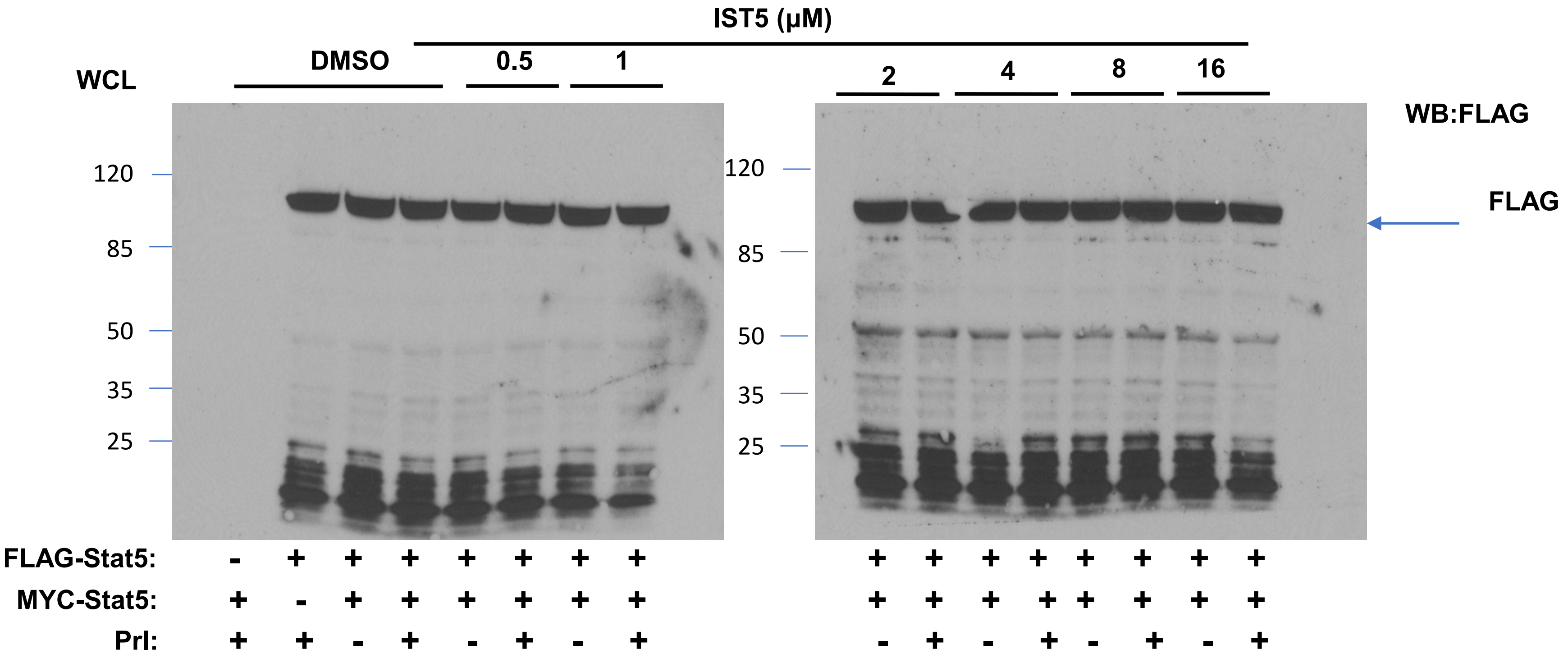
IST5 (μM)
WCL
DMSO
0.5
1
2
4
8
16
WB:FLAG
FLAG
120
85
50
35
25
FLAG-Stat5: - + + + + + + + + + + + + + + +
MYC-Stat5: + - + + + + + + + + + + + + + +
Prl: + + - + - + - + - + - + - + - +

Figure 5B

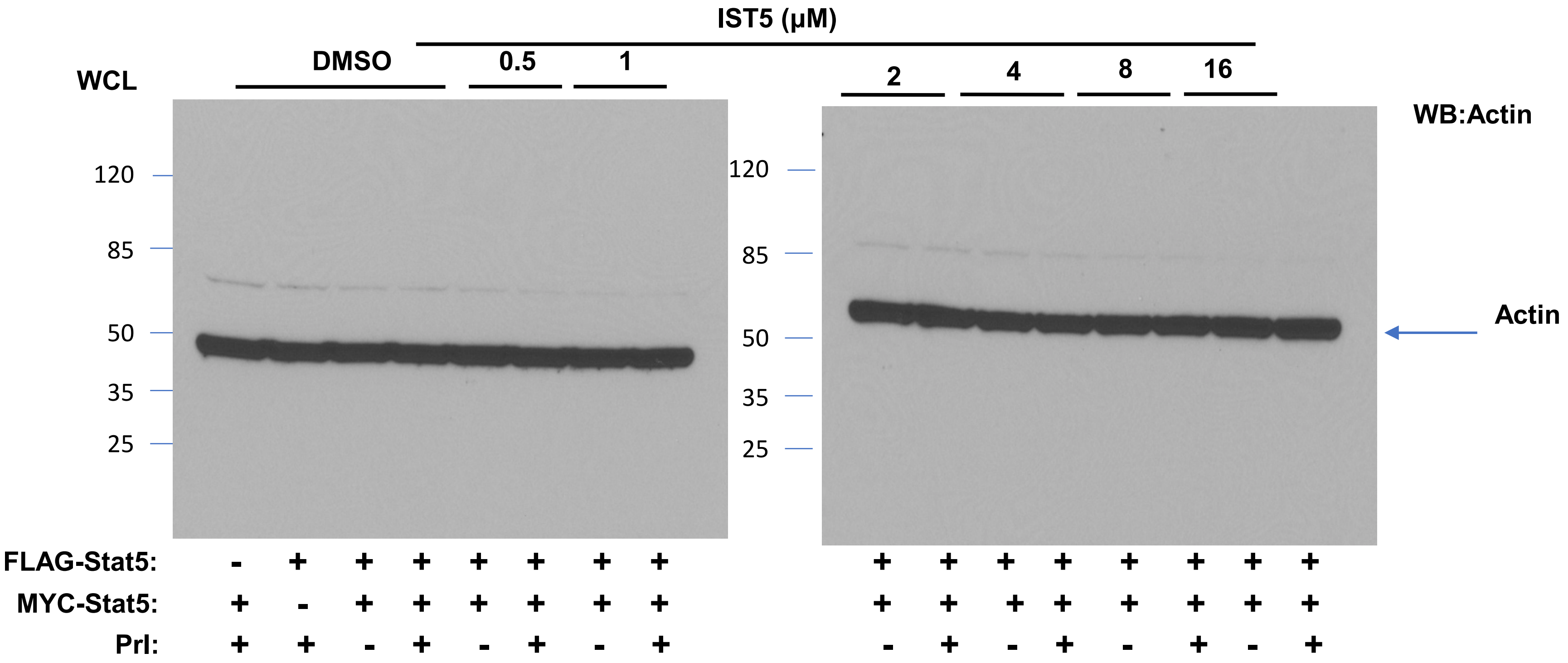
IST5 (µM)
WCL
DMSO
0.5
1
2
4
8
16
WB:Actin
120
85
50
35
25
Actin
FLAG-Stat5:
MYC-Stat5:
Prl:

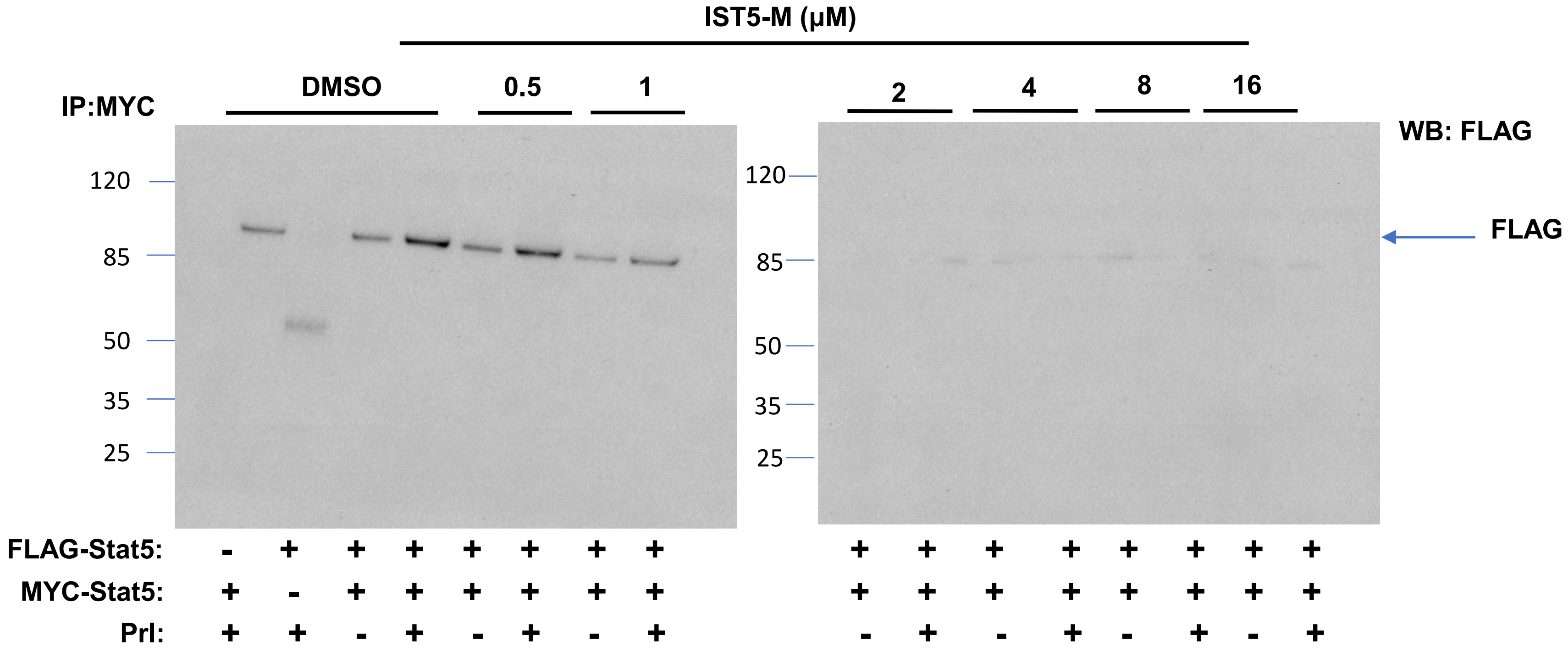
IST5-M (µM)
IP:MYC
DMSO
0.5
1
2
4
8
16
WB: FLAG
FLAG
120
85
50
35
25
FLAG-Stat5:
MYC-Stat5:
Prl:

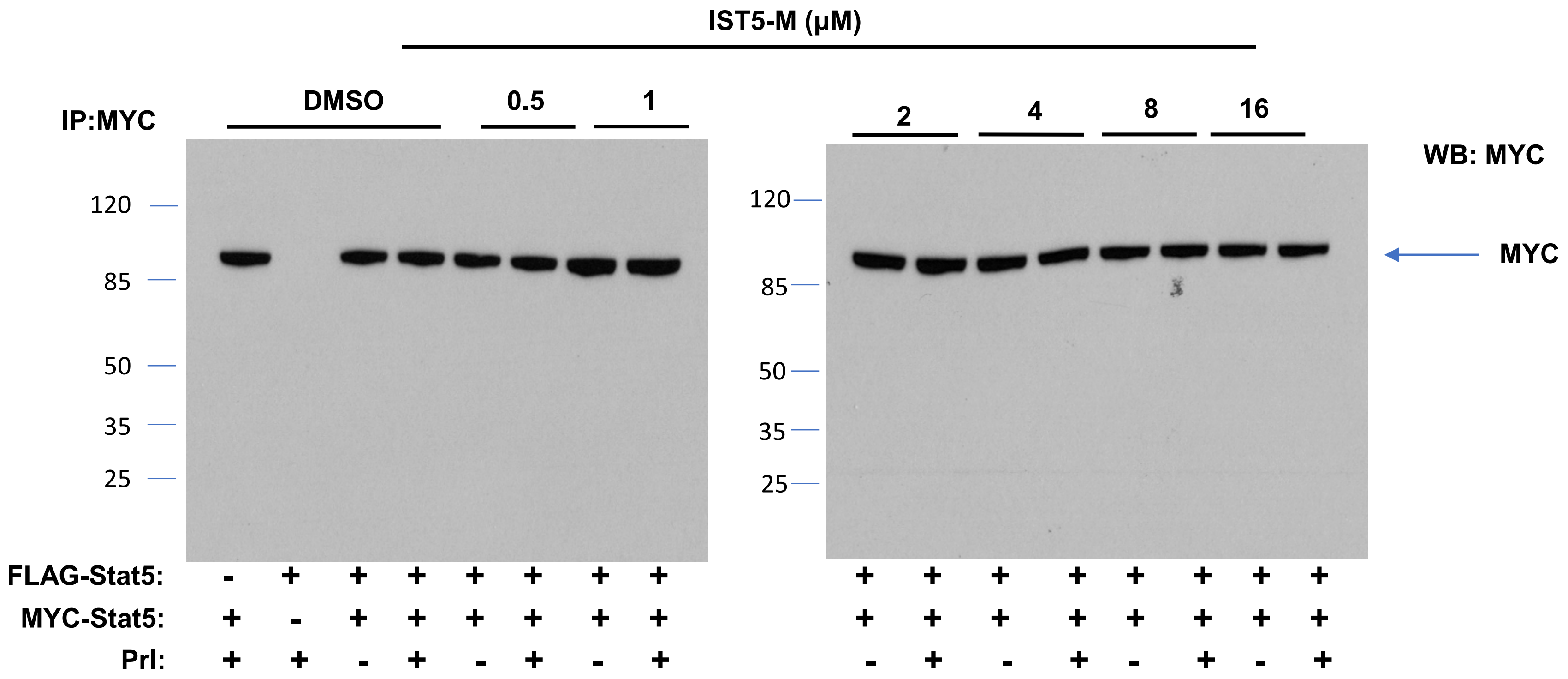
IST5-M (μM)
IP:MYC
DMSO
0.5
1
2
4
8
16
WB: MYC
120
85
50
35
25
MYC
FLAG-Stat5: - + + + + + + + + + + + + + + +
MYC-Stat5: + - + + + + + + + + + + + + + +
Prl: + + - + - + - + - + - + - + - +

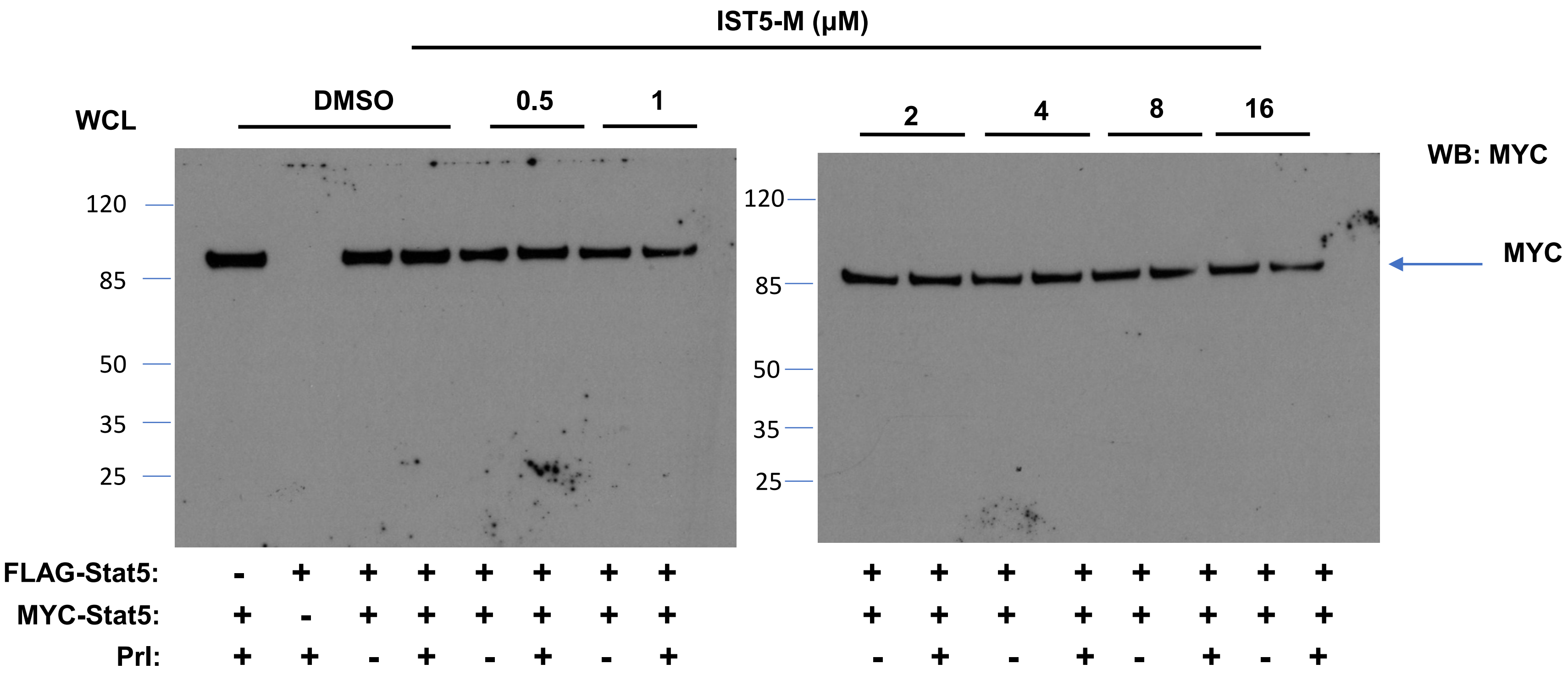
IST5-M (µM)
WCL
DMSO
0.5
1
2
4
8
16
WB: MYC
120
85
50
35
25
120
85
50
35
25
MYC
FLAG-Stat5: - + + + + + + + + + + + + + + +
MYC-Stat5: + - + + + + + + + + + + + + + +
Prl: + + - + - + - + - + - + - + - +

Figure 5B

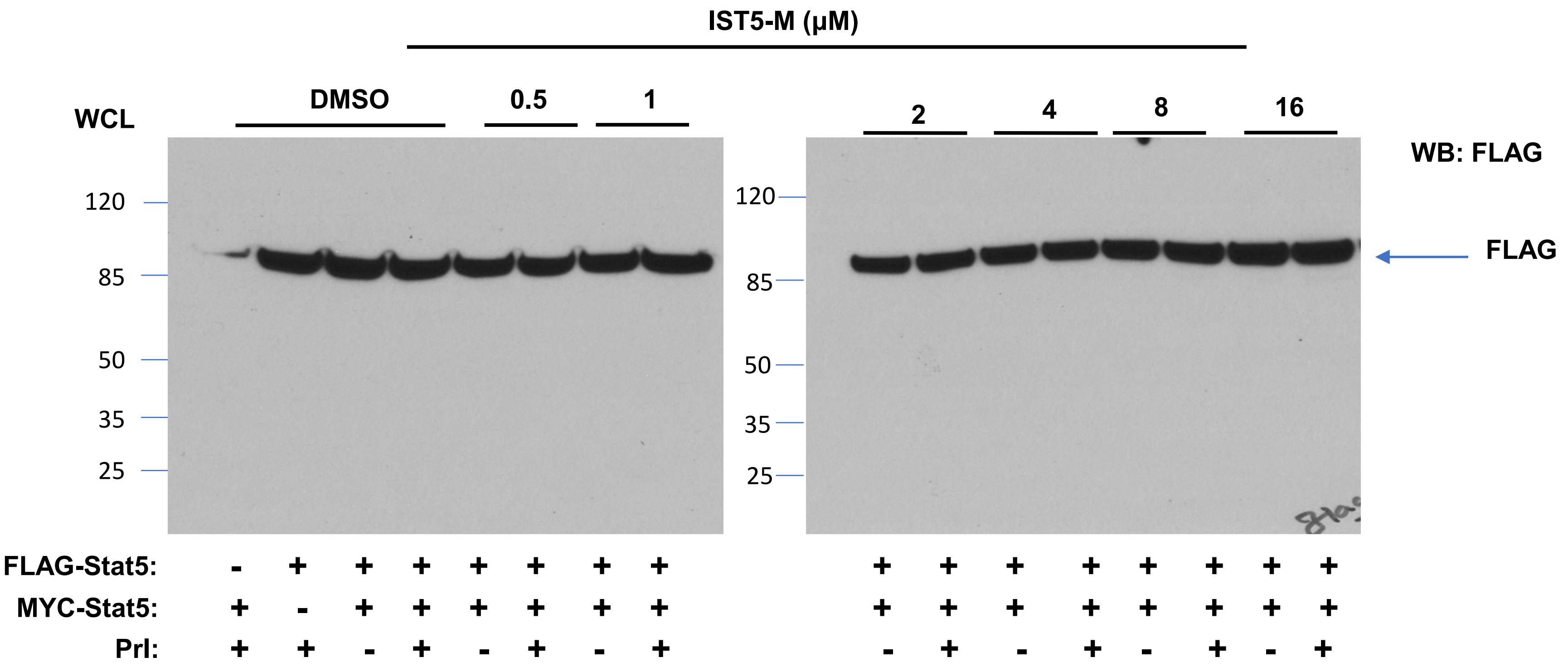
IST5-M (µM)
WCL
DMSO
0.5
1
2
4
8
16
WB: FLAG
120
85
50
35
25
120
85
50
35
25
FLAG
FLAG-Stat5: - + + + + + + + + + + + + + + +
MYC-Stat5: + - + + + + + + + + + + + + + +
Prl: + + - + - + - + - + - + - + - +

Figure 5B

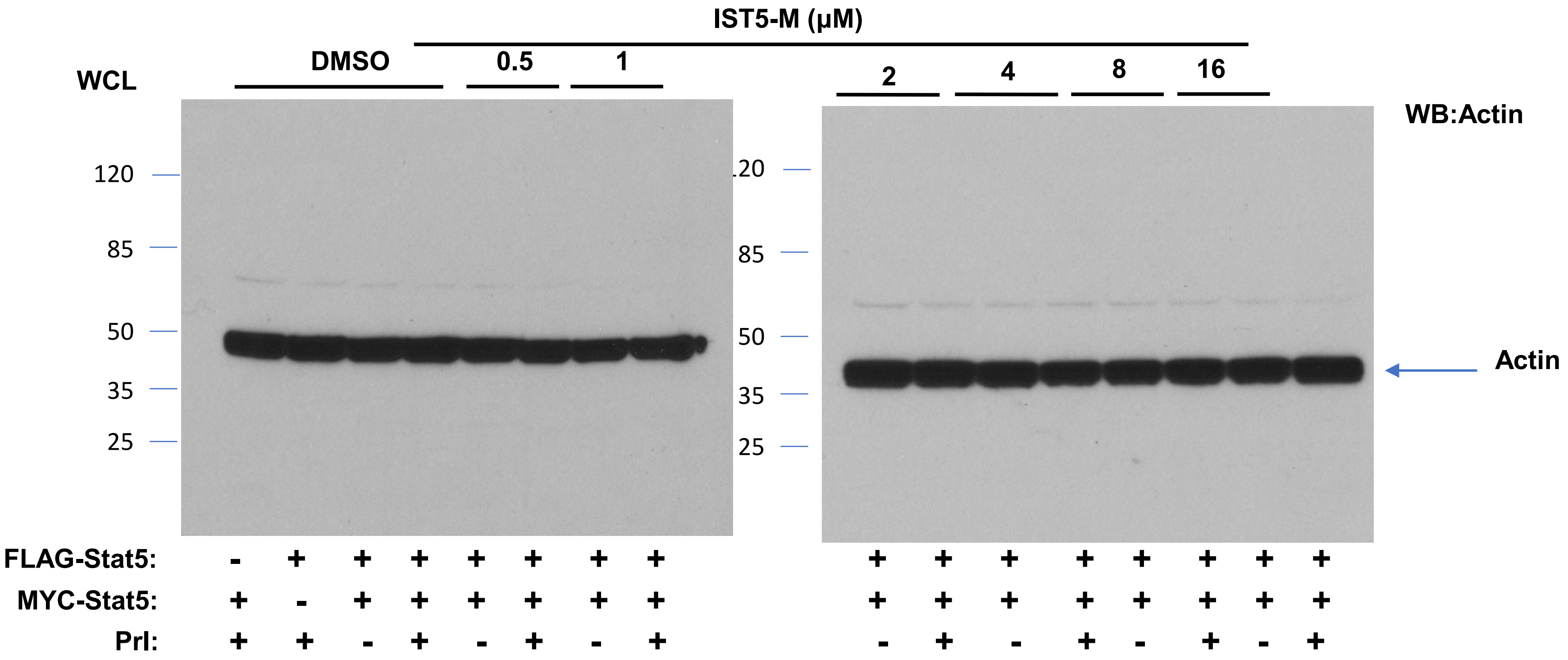
IST5-M (μM)
WCL
DMSO
0.5
1
2
4
8
16
WB:Actin
120
85
50
35
25
20
85
50
35
25
Actin
FLAG-Stat5: - + + + + + + + + + + + + + + +
MYC-Stat5: + - + + + + + + + + + + + + + +
Prl: + + - + - + - + - + - + - + - +

Figure 5D

DU145

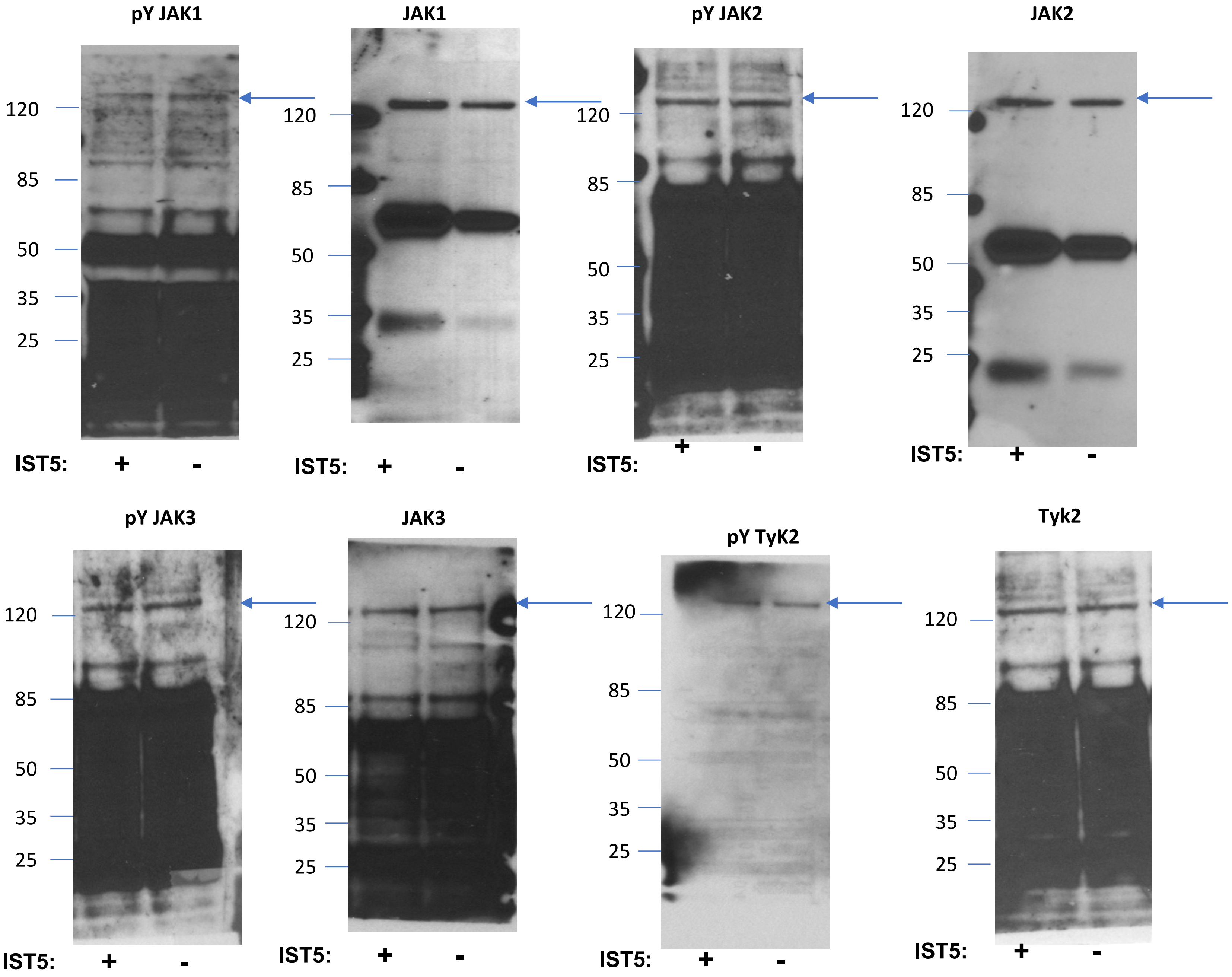

Figure 5D

CWR22Pc

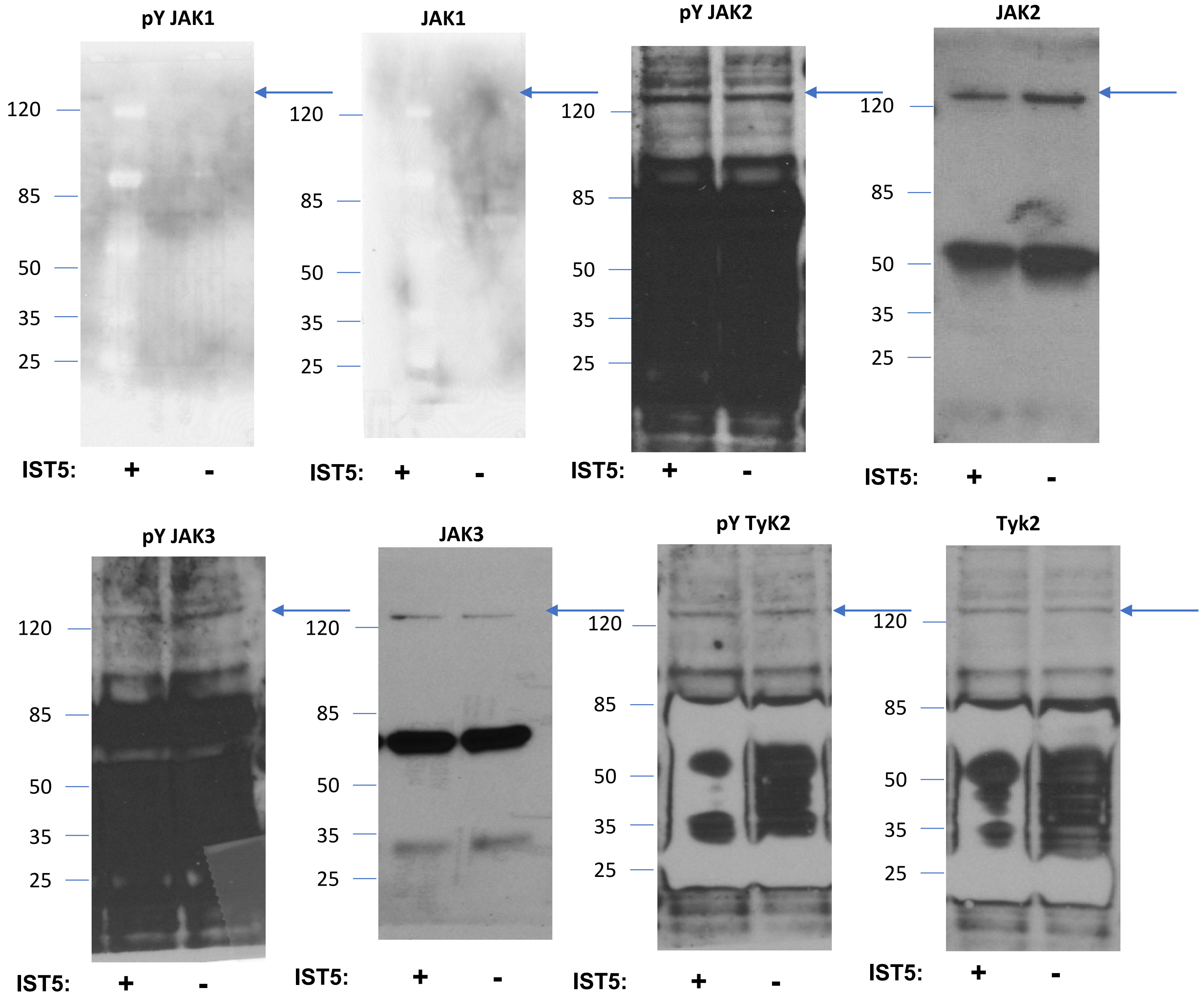

Native Coomassie gels

Figure 5A Left Panel

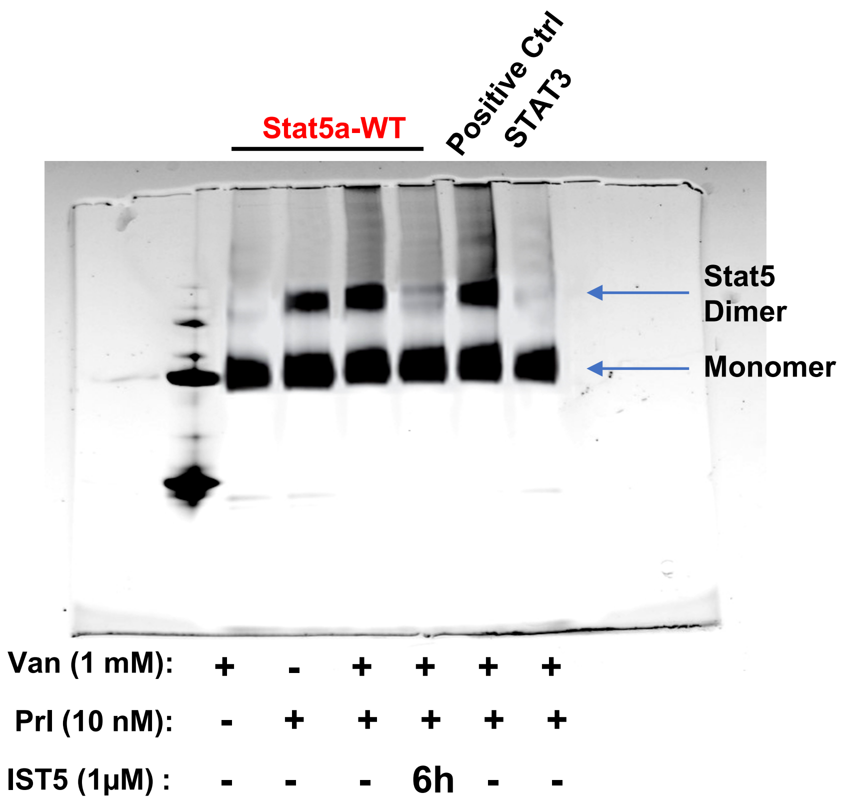

Figure 5A Right Panel

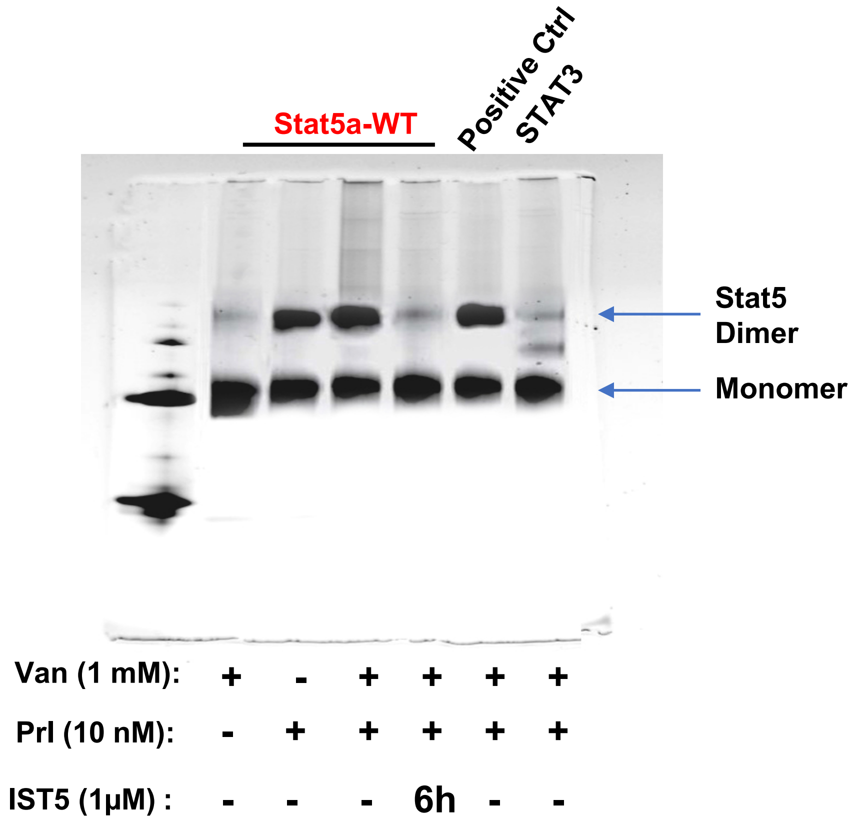

Supplement: Supplementary file 1 [file cancers-12-03412-s001.zip › cancers-997294-supplementary/cancers-997294-WB.pdf]
